# ACSL1 Aggravates Thromboinflammation by LPC/LPA Metabolic Axis in Hyperlipidemia Associated Myocardial Ischemia‐Reperfusion Injury

**DOI:** 10.1002/advs.202406359

**Published:** 2025-01-23

**Authors:** Shuai Jiang, Xueguang Lin, Bo Chen, Gang Chen, Kristine J.S. Kwan, Jing Liu, Qi Sun, Jie Wang, Yijie Lu, Jindong Tong, Ying Deng, Bo Yu, Jingdong Tang

**Affiliations:** ^1^ Shanghai Key Laboratory of Vascular Lesions and Remodeling Department of Vascular Surgery Shanghai Pudong Hospital Fudan University Pudong Medical Center Shanghai 201399 China; ^2^ Department of Cardiology Shanghai Pudong Hospital Fudan University Pudong Medical Center Shanghai 201399 China; ^3^ State Key Laboratory of Genetic Engineering Collaborative Innovation Center for Genetics and Development School of Life Sciences and Human Phenome Institute Fudan University Shanghai 200438 China; ^4^ Department of Endocrinology and Metabolism Affiliated Hospital of Nantong University Nantong 226006 China; ^5^ Department of Vascular Surgery Huashan Hospital Fudan University Shanghai 200040 China

**Keywords:** ACSL1, hyperlipidemia, ischemia‐reperfusion injury, lipid metabolism, thromboinflammation

## Abstract

Acute myocardial infarction (AMI) is associated with well‐established metabolic risk factors, especially hyperlipidemia and obesity. Myocardial ischemia‐reperfusion injury (mIRI) significantly offsets the therapeutic efficacy of revascularization. Previous studies indicated that disrupted lipid homeostasis can lead to lipid peroxidation damage and inflammation, yet the underlying mechanisms remain unclear. Here, the study demonstrates that hyperlipidemia is a key driver of mIRI. Long‐chain fatty acyl‐CoA synthetase 1 (ACSL1) is upregulated in both hyperlipidemia and AMI patients. ACSL1 expression is induced by a high‐fat microenvironment (oxLDL and palmitic acid) in a concentration‐dependent manner. Interestingly, the protein level is positively correlated with total cholesterol level and thromboinflammatory biomarkers. Furthermore, ACSL1 reprogrammed lipid metabolism in monocytes, leading to the accumulation of lysophosphatidylcholine (LPC)/lysophosphatidic acid (LPA). The monocytic LPC/LPA axis accelerated lipid peroxidation and neutrophil extracellular traps (NETs)‐induced thromboinflammation via the paracrine effect. The main LPA producer Autotaxinis is also induced under high‐fat conditions and then exerts thromboinflammation response through converted LPC to LPA. Finally, ACSL1 knockdown or NETs release inhibitor (DNase I or GSK484) significantly alleviated mIRI in mice. These findings highlight ACSL1 and NETosis as potential key targets for preventing mIRI and underscore the lipid peroxidation in the mechanisms of ACSL1‐mediated thromboinflammation.

## Graphical abstract

In hyperlipidemic states, Long‐chain fatty acyl‐CoA synthetase 1 (ACSL1) is consistently induced, which then reprogrammes the lipid metabolism in monocytes. The accumulation of monocyte‐secreted lysophosphatidylcholine (LPC)/lysophosphatidic acid (LPA) enhances the thrombotic and inflammatory processes Then LPC/LPA axis promots activation and NETosis in neutrophils in a paracrine‐dependent manner. Finally, this leads to myocardial ischemia‐reperfusion injury‐related thromboinflammation response.

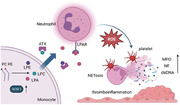

## 1 Introduction

Acute myocardial infarction (AMI) denotes the sudden rupture of atherosclerotic coronary plaques that induces the death of cardiomyocytes due to ischemia.^[^ ^1^ ^]^ Reperfusion therapies have led to a substantial reduction in the frequency of mechanical complications of AMI. Prompt reperfusion may potentially salvage the ischemic myocardium but paradoxically cause myocardial ischemia‐reperfusion injury (mIRI) by permitting the process of thromboinflammation.^[^ ^2^ ^]^

The incidence of AMI continues to increase globally, growing evidence shows hyperlipidemia, obesity, and sedentary living are the main metabolic risk factors.^[^ ^3^ ^]^ Patients with familial hypercholesterolemia has genetically suffering from an early incident and 2.5‐times higher risk of recurrent AMI event.^[^ ^4^ ^]^ High‐fat environments facilitated lipid peroxidation when reactive oxide species (ROS) existed. Lipid peroxidation could be the underly driver that accelerates atherothrombosis and eventually leads to AMI.^[^ ^5^ ^]^ The fact that hyperlipidemia is associated with increased lipid peroxidation and greater consumption of antioxidants is well established.^[^ ^6^ ^]^ Additionally, hyperlipidemia impairs the clearance of neutrophil extracellular traps (NETs) and promotes inflammation.^[^ ^7^ ^]^ Therefore, identifying the relationship between the hyperlipidemic environment and NETs in the context of mIRI may offer insight into a new treatment strategy without directly eliminating NETs and impairing the innate immune defense.

In this study, key genes involved in the aggravation of mIRI were identified by comprehensive data mining and validation. Overexpression of ACSL1 was validated by in vivo mIRI mice models and serum samples from clinical patients. Subsequently, we further investigated the influence of ACSL1 on monocyte‐neutrophil interplay and found that ACSL1 overexpression accelerated NETs release, thereby promoting thromboinflammation through the LPC/ ATX/LPA axis. This study boldly explores the metabolic mechanisms building on previous observations that dyslipidemia exacerbates IRI.

## 2 Results

### 2.1 Increased ACSL1 Expression in Patients with Hyperlipidemia and Acute Myocardial Infarction

**Figure 1:**
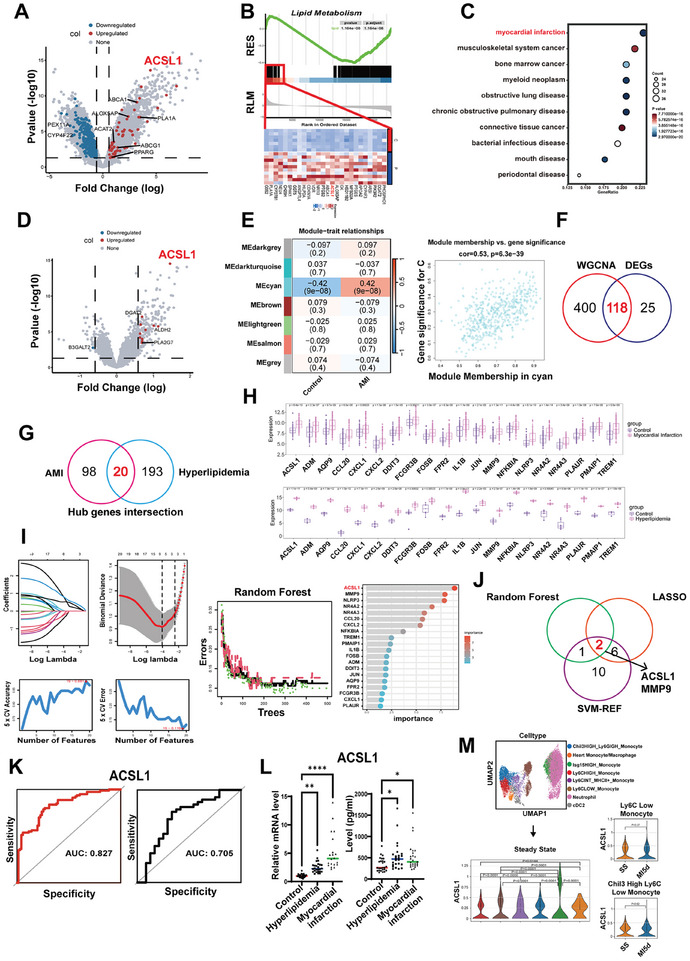
ACSL1 expression is commonly upregulated in patients with hyperlipidemic conditions or AMI. Bulk RNAseq was performed in hyperlipidemia patients (*n* = 10) and healthy controls (*n* = 7). A) Volcano plot of DEGs of PBMCs followed the criteria of *p* < 0.05 and |log2FoldChange|> 1. B) GSEA of lipid metabolism‐related genes. Top panel: overrepresentation of lipid metabolism genes (p‐value = 1.16e‐08, adjusted). Middle panel: Ranked metric scores with key genes highlighted. Bottom panel: Heatmap of gene expression in lipid metabolism pathway. C) Disease ontology (DO) analysis based on the hyperlipidemia‐related hub genes. Then public datasets were analyzed, D) Volcano plot; E) WGCNA module‐trait correlation heatmap and scatter plot; F) Venn diagram; G) Venn diagram; H) Box plots comparing the expression levels (FPKM) of 20 key genes shared between acute myocardial infarction (AMI) and hyperlipidemia across different patient groups (*t*‐test). Additionally, I) Comprehensive analysis of comorbidity genes was screened by three machine learning approaches: LASSO regression, univariate Cox regression, and Random Forest. J) Venn diagram illustrating gene overlaps of comorbidity genes. K) ROC curves for ACSL1 demonstrating diagnostic performance. Finally, ACSL1 expression was validated L) in PBMCs and serum samples of patients with hyperlipidemia and AMI compared to controls (For qRT‐PCR analysis, control, *n* = 25; hyperlipidemia, *n* = 31; AMI, *n* = 21. For ELISA, control, *n* = 26; hyperlipidemia, *n* = 24; AMI, *n* = 25. one‐way ANOVA) and M) Single‐cell RNA sequencing analysis of mouse heart cells. ^*^ *p* < 0.05, ^**^ *p* < 0.01, and ^***^ *p* < 0.001.

To identify and validate pivotal genes implicated in hyperlipidemic conditions and AMI, a comprehensive series of investigations were conducted. Initially, bulk RNA‐sequencing was performed on peripheral blood mononuclear cells (PBMCs) collected from 10 hyperlipidemic patients and 7 healthy controls. A total of 1696 differentially expressed genes (DEGs) with 679 upregulated in AMI patients, using stringent filtering criteria (Padj < 0.05 and |log2FoldChange| > 1). Notably, metabolic DEGs such as ACSL1, ABCA1, ABCG1, ACAT2, ALOX5AP, PLA1A, and PPARG were elevated in hyperlipidemia (**Figure** **1**A). The Gene Set Enrichment Analysis (GSEA) of lipid metabolism pathways also demonstrated that significant overrepresentation of lipid metabolism‐related genes in hyperlipidemia (p‐value = 1.16e‐08) (Figure 1B). Disease Ontology (DO) analysis further suggested significant involvement of DEGs in cardiovascular diseases, particularly myocardial infarction (Figure 1C). Additionally, we conducted a protein–protein interaction (PPI) network analysis of the hyperlipidemia dataset. The 213 hub DEGs were significantly enriched in response to reactive oxygen species, lipid and atherosclerosis, and so on (Figure S1a–c, Supporting Information).

Given the DO analysis indicating DEGs' enrichment in myocardial infarction, we selected GEO datasets GSE48060 and GSE66360 relevant to AMI, for further investigation. ACSL1 was also one of the top upregulated metabolic DEGs in AMI (Figure 1D). Weighted Gene Co‐expression Network Analysis (WGCNA) was performed on 21654 genes from 151 samples, producing a cluster dendrogram with cyan modules that exhibited a strong correlation with AMI (Figure 1E). From this analysis, 118 overlapping DEGs were identified between AMI DEGs and cyan module hub genes (Figure 1F). The Venn diagram depicts the intersection of hub genes identified from PPI analysis of hyperlipidemia data and key genes previously identified in AMI, revealing 20 shared key genes associated with both conditions (Figure 1G,H). Then three distinct machine learning algorithms—random forest, SVM‐RFE, and LASSO—were applied for further screening. We found that ACSL1 and MMP9 were pivotal genes common to both hyperlipidemic and AMI conditions (Figure 1I,J). ACSL1 demonstrated robust diagnostic and predictive value, as indicated by ROC curve analysis (AUC = 0.827; Figure 1K). Subsequent analysis of ACSL1 in the AMI dataset utilizing gene set variation analysis (GSVA) elucidated its association with various biological pathways. Heatmaps illustrating GSVA‐based expression levels and pathway association scores for ACSL1 across diverse biological pathways revealed significant correlations of ACSL1 with key pathways related to lipid metabolism and inflammatory responses, with red indicating higher activity or positive correlation and blue indicating lower activity or negative correlation (Figure S1g, Supporting Information).

We also validated the high expression of ACSL1 in the AMI population through both internal and external cohorts (Figure S1d–f, Supporting Information). Additionally, we prospectively recruited 76 patients, comprising 25 control subjects, 31 hyperlipidemia patients, and 21 AMI patients for qRT‐PCR analysis. 26 control subjects, 24 hyperlipidemia patients, and 25 AMI patients for ELISA. Elevated levels of ACSL1 expression were detected in their serum and PBMC samples (Figure 1L), corroborating the bioinformatics analysis findings. Finally, single‐cell RNA sequencing analysis of mouse heart cells also depicted ACSL1 expression levels across different monocyte subsets and macrophages, revealing variability within and between these cell types (Figure 1M).

### 2.2 ACSL1 Correlates to NETs‐Induced Thromboinflammation

**Figure 2:**
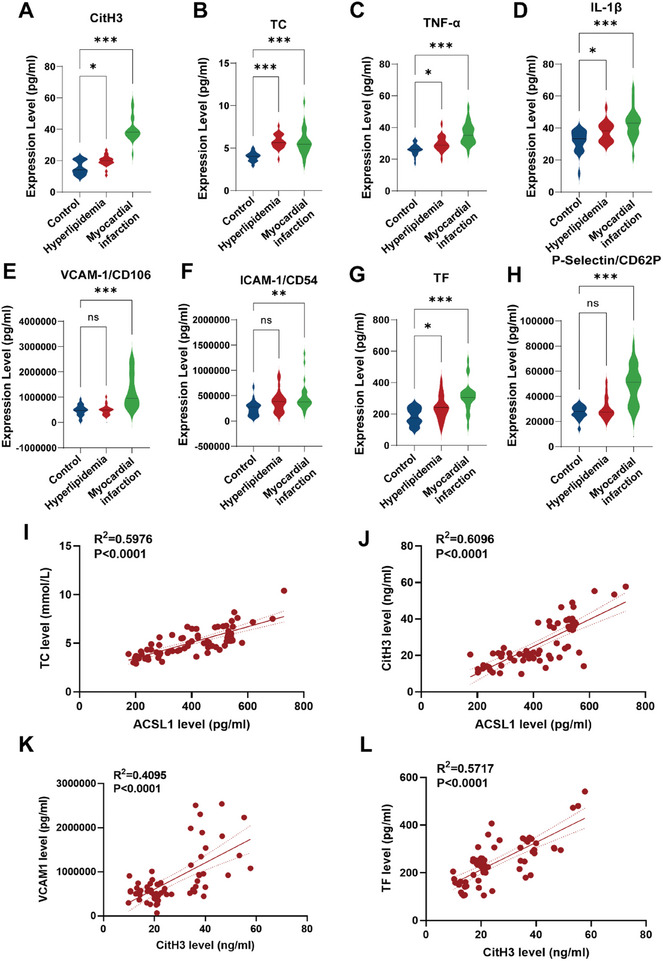
Increased circulating levels of thrombo‐inflammation markers in hyperlipidemia and AMI patients were related to ACSL1. A–H) CitH3 (A), TC (B), TNFα (C), IL‐1β (D), VCAM‐1/CD106 (E), ICAM‐1/CD54 (F), TF (G), P‐selectin/CD62P (H) were measured by multiple Luminex assay. (Control: *n* = 28; Hyperlipidemia: *n* = 28; Myocardial Infarction: *n* = 26. one‐way ANOVA). I,J) Correlation analysis showing the relationships between serum ACSL1 levels and clinical biomarkers in patients. K,L) Correlation analysis between CitH3 levels and inflammatory biomarkers VCAM‐1 and TF in patients. ^*^p < 0.05, ^**^ *p* < 0.01, and ^***^ *p* < 0.001 versus control.

Given that thrombosis and inflammation were the main signatures of AMI, we enrolled 69 patients and 77 healthy controls, with baseline demographics detailed in Table S1 (Supporting Information). We found that CitH3, Elastase, total cholesterol (TC), TNF‐α, IL‐1β, VCAM‐1, ICAM‐1, tissue factor (TF), and P‐selectin were significantly increased in hyperlipidemia, and further increased in AMI patients (**Figure** **2**A–H; Figure S2a–f, Supporting Information). The cardiac marker cardiac troponin (Tn) was also increased in hyperlipidemia and AMI patients, with a significantly positive relationship between ACSL1 levels and cardiac markers in patients (Figure S2g,h, Supporting Information). Correlation analysis demonstrated that ACSL1 levels were strongly associated with both blood lipid levels and thromboinflammatory biomarkers. For instance, ACSL1 showed a significant correlation with total cholesterol (TC) (R^2^ = 0.5976, P < 0.0001) and CitH3 (R^2^ = 0.6096, P < 0.0001) (Figure 2I,J). Additionally, CitH3 levels were correlated with key coagulation and inflammatory biomarkers, including VCAM‐1 (R^2^ = 0.4095, P < 0.0001) and TF (R^2^ = 0.5717, P < 0.0001) (Figure 2K,L), thus confirming the association between NETs formation and thromboinflammation.

NETs formation is recognized as a key mechanism underpinning thromboinflammation.^[^ ^8^ ^]^ We observed elevated serum elastase levels in both AMI and hyperlipidemic patients (Figure S3a, Supporting Information). Flow cytometry analysis revealed a higher proportion of activated neutrophils (CD45^+^CD66b^+^CD16^+^) in the PBMCs of AMI patients (Figure S3b,c, Supporting Information). The abundance of NET‐forming neutrophils, characterized by MPO^+^CitH3^+^ events, indicated that NETs‐induced thromboinflammation played a significant role in AMI pathogenesis and was closely associated with elevated ACSL1 expression.^[^ ^9^ ^]^ This comprehensive analysis suggests that ACSL1 is not only a pivotal gene linking hyperlipidemia and AMI but also a key player in the thromboinflammatory processes that characterize these conditions.

### 2.3 ACSL1‐Dependent Monocyte Activation Accelerates Thromboinflammation in Hyperlipidemia

**Figure 3:**
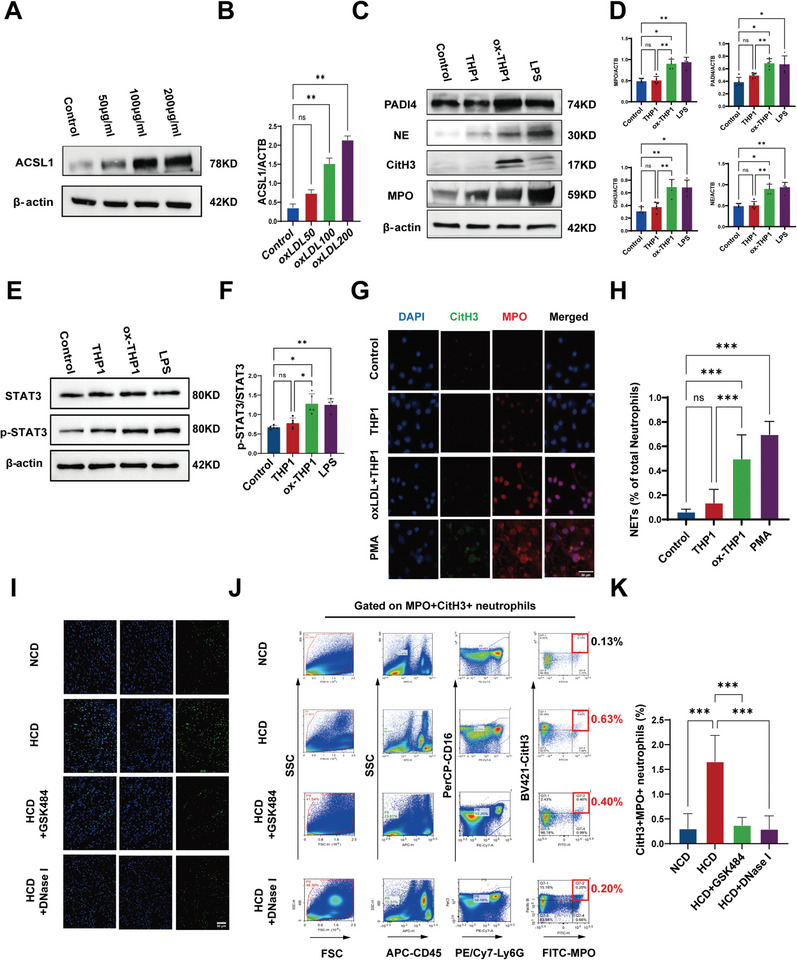
Hyperlipidemia accelerates thromboinflammation in vitro and in vivo via ACSL1‐dependent monocyte activation. A,B) Immunoblotting and quantification showing the expression of ACSL1 in THP1 after stimulation by 50/100/200 µg mL^−1^ oxLDL for 24 h. The stripes were analyzed by Image J software (*n* = 3). C,D) Expression and quantification of NETs‐related and neutrophil activation markers in peripheral neutrophils from patients, either stimulated with LPS or co‐cultured with THP1 monocytes that were pre‐treated with or without 100 µg mL^−1^ oxLDL. The stripes were analyzed by Image J software (*n* = 5). E,F) Western blot analysis of STAT3 and p‐STAT3 in human neutrophils exposed to control conditions, cocultured with THP1 cells, THP1 stimulated by oxLDL, and LPS. The stripes were analyzed by Image J software (*n* = 5). G) Representative immunofluorescence staining images of CitH3 (green) and MPO (red) DAPI (blue) in peripheral neutrophils exposed to PMA (250 ng mL^−1^) for 3 h or co‐cultured with THP1 monocytes that were pre‐treated with or without 100 µg mL^−1^ oxLDL. H) Quantification of the proportion of NETosis, identified by CitH3^+^MPO^+^ staining, in all neutrophils. (*n* = 10 in each group). Scale bar: 30 µm. I) Apoptotic cells in heart tissue from one mouse in each group are displayed. The quantification of TUNEL‐positive cells around the infarct area is presented. (*n* = 5 in each group; mean ± S.D; ^**^ *p* < 0.01, ^***^ *p* < 0.001 versus ApoE^−/−^ (NCD) + vehicle). Scale bar: 0 µm. J,K) Representative flow cytometry density plots of fresh blood [quantified in (K) panel; *n* = 5 in each group]. Data were shown as mean ±S.D from at least three independent experiments, one‐way ANOVA. ^**^ *p* < 0.01, ^***^ *p* < 0.001 versus control group.

To identify the main dysregulated cell type in the pathogenesis. CIBERSORT analysis showed a robust correlation between ACSL1 and both monocytes and neutrophils (Figure S4, Supporting Information). So oxLDL and palmitic acid (PA) were used to stimulate THP1 cells thereby creating a high‐fat cell (HFC) model.^[^ ^10^ ^]^ ACSL1 protein and mRNA expression levels were significantly increased in oxLDL/PA‐treated THP1 cells in a concentration‐dependent manner (**Figure** **3**A,B; Figure S5a,b, Supporting Information). This treatment led to a notable augmentation in the presence of immune cells and crucial thromboinflammation‐associated cellular components.

To further understand the interplay between monocytes and neutrophils under hyperlipidemic conditions, we employed a transwell system to co‐culture isolated human primary neutrophils with THP1 cells stimulated by oxLDL/PA. We found overexpression of ACSL1 could significantly increase total and mitochondrial ROS production, but with no influence on cell viability and apoptosis (Figure S5c–g, Supporting Information). Surprisingly, increased markers of neutrophil activation and NETosis, such as phosphorylated STAT3, CitH3, neutrophil elastase (NE), PADI4, and dsDNA, were detected in neutrophils stimulated by HFCs (Figure 3C–H). However, no significant difference was observed between direct contact co‐culture and co‐culture using transwell inserts, suggesting that surface adhesion molecules were not implicated in this process (Figure S5h, Supporting Information).^[^ ^11^ ^]^ These findings indicate that high‐fat monocytes can trigger neutrophil activation independently of direct cell contact.

Then we also found that stable knockdown ACSL1 or pan‐ACSL1 inhibitor (Triacsin C) could significantly decrease neutrophil NET formation (Figure S6a–d, Supporting Information). Conversely, NETosis increased among neutrophils co‐cultured with a stable THP1 cell line overexpressing ACSL1, irrespective of high‐fat stimulation (Figure S6e,f, Supporting Information). Similar results were obtained by replacing the conditioned media (CM) of co‐cultured THP1 cells (Figure S7a–e, Supporting Information).

Moreover, comorbidity mouse models with hyperlipidemia and mIRI were prepared in ApoE^−/−^ background mice. Mice were fed with a high‐cholesterol diet (HCD) or a normal diet (NCD). We found that significantly worse severity of mIRI and heightened NETosis activity in a HCD group (Figure 3I; Figure S8a,b, Supporting Information). The severity of myocardial infarction was notably reduced by DNase I and GSK484 treatment, highlighting the role of NETosis in exacerbating mIRI. Platelet‐neutrophil complexes (PNCs) serve as markers in various inflammatory and cardiovascular disorders.^[^ ^12^ ^]^ Flow cytometry analysis revealed higher ratios of NET‐producing neutrophils in peripheral blood samples from HCD‐fed mice, characterized by CD45^+^Ly6G^+^CD11b^+^ activation. These ratios decreased upon treatment with NETosis inhibitors including GSK484 or DNase I (Figure 3J,K).

In summary, hyperlipidemia accelerates thromboinflammation in vitro and in vivo via ACSL1‐dependent monocyte activation, further demonstrating that NETosis is accelerated in hyperlipidemic states, which consequently aggravates the thromboinflammatory process in mIRI. This phenomenon was also observed in patient samples, establishing ACSL1 as a crucial player in the pathogenesis of hyperlipidemia and AMI.

### 2.4 ACSL1 Overexpression Promoted Metabolic Reprogramming of Monocytes

**Figure 4:**
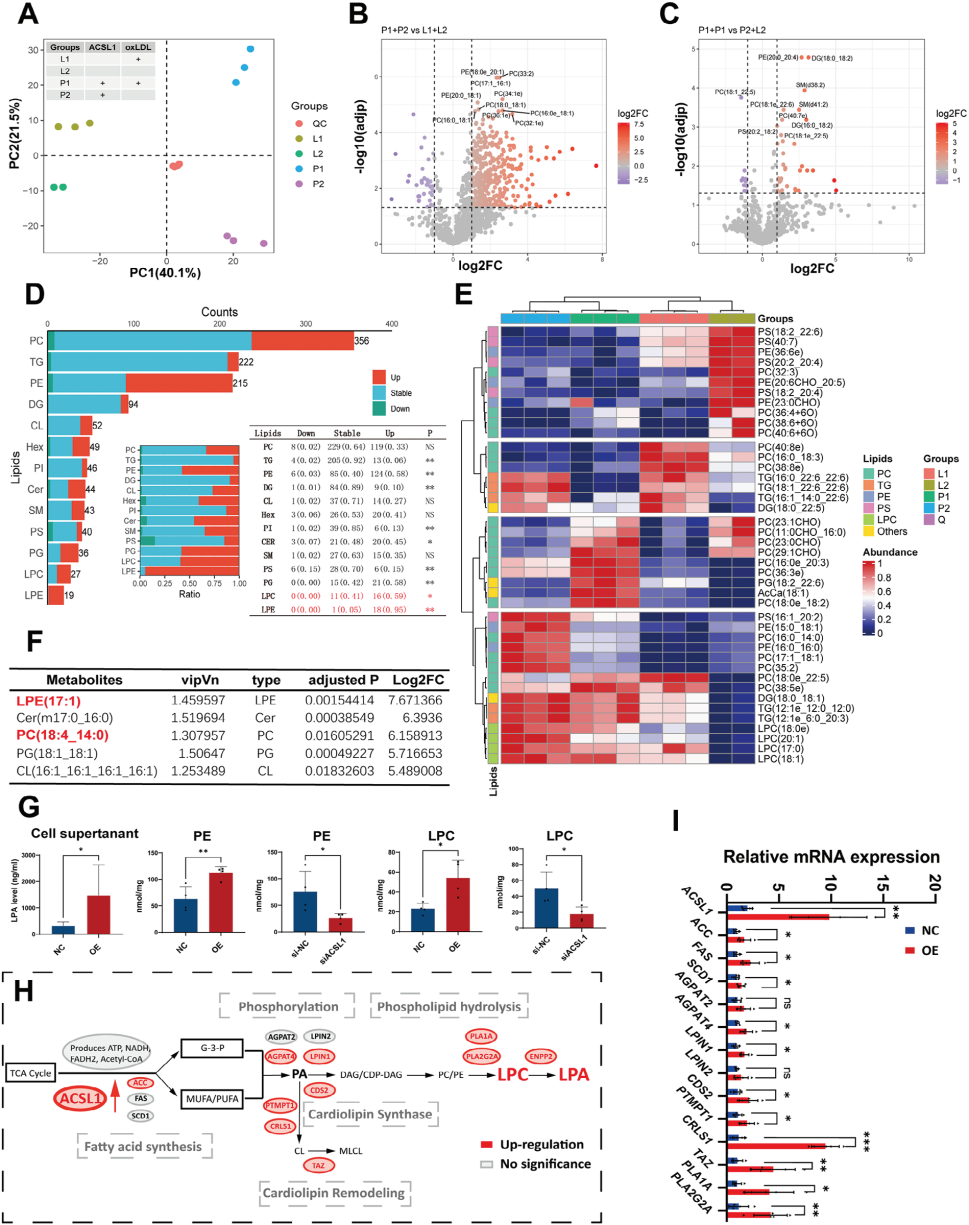
Overexpression of ACSL1 alters THP‐1 monocyte's lipidome. A) Principal component analysis (PCA) of all metabolome samples. ACSL1 overexpressed group: P1+P2; control group1: L1+L2; oxLDL treated group: P1+L1; control group2: P2+L2; P1+P2 and L1+L2 are distinctly separated on PC1, accounting for 40.1% of the variance in the original data; P1+L1 and P2+L2 are significantly differentiated on PC2, explaining 21.5% of the variance in the original data. B,C,E) Volcano plots and heatmap showing the results of pairwise comparisons of metabolites in oxLDL treated group (lower) and ACSL1 overexpressed group (upper left) relative to controls (B, ACSL1 overexpressed group versus control; C, oxLDL treated group versus control). log2FC > 1, adjusted *p* < 0.05. D) Relative change, along with the proportion of upregulated, downregulated, and unchanged metabolites, identified in THP1 monocytes overexpressing ACSL1 compared to control THP1 monocytes. F) The metabolites exhibited the greatest fold change in the ACSL1 overexpression group relative to the control group. G) Assay kit of LysoPC and PE in ACSL1 overexpressed and control THP1 monocytes. (*n* = 4 samples in each group; mean ± S.D; ^*^ *p* < 0.05, ^**^ *p* < 0.01 versus control). H,I) Schematic illustration (H) of genes involved in de novo LysoPE/LysoPC/LysoPA synthesis and quantitative RT‐PCR (I) of genes involved in lipogenesis and GPL synthesis based on lipid metabolome data. (*n* = 4 samples in each group; mean ± S.D; ^*^ *p* < 0.05, ^**^ *p* < 0.01 versus control).

To explore the modulation function of ACSL1 on lipid metabolism, a mass spectrometry‐based lipidomic analysis was conducted in ACSL1 overexpressed THP1 cell lines treated with or not with oxLDL. Principal component analysis (PCA) revealed a clear clustering of samples among conditions, indicative of extensive metabolite changes (**Figure** **4**A). Overall, the changes in lipid levels due to ACSL1 modulation were more significant than the effects seen with or without oxLDL (Figure 4B,C). In contrast to oxLDL‐treated groups, ACSL1 demonstrated a prominent contribution to lipid metabolism reprogramming of THP1 cells by 32% of lipid levels (log2FC > 1; adjusted *p* < 0.005). The main differentially expressed lipids were lysophosphatidylethanolamine (LPE; 94.7%), LPC (59.3%), cardiolipin (CL; 26.9%), and phosphatidylcholine (PC; 22.4%) when compared to oxLDL‐treated controls (Figure 4D,E). Elevated amounts of single unsaturated fatty acids (FAs), including LPE (17:1), LPE (16:1), CL (16:1)4, CL (17:1/16:1/16:1/18:1), and LPC (16:1), was found in ACSL1 overexpressed THP1 cells when compared to control (Figure 4F). These phospholipids and their respective derivatives participate in pathological processes associated with NETs and thrombosis.^[^ ^13^ ^]^

We further validated these metabolites in cell supernatant and reported their absolute levels in cell homogenate (Figure 4G). Additionally, abnormal mRNA levels of enzymes associated with metabolite metabolism in key molecular pathways were also verified, where ACSL1 affects de novo lipidogenesis (Figure 4H,I).^[^ ^14^ ^]^ In the extracellular space, ATX hydrolyzes LPC to generate LPA, which is a NETs induction metabolite that induces NETs.^[^ ^13a^ ^]^ We observed elevated LPA in CM of cultured ACSL1 overexpressed THP1 monocytes (Figure 4g). Thereby suggesting that lipids released by THP1 monocytes undergoing ACSL1‐dependent lipidomic remodeling could serve as substrates for the synthesis of NETs induction lipids, facilitated by ATX‐mediated LPC hydrolysis.

### 2.5 ACSL1 Overexpression Upregulates of LPC‐ATX‐LPA Axis in Monocytes and Potentiates NETs‐Induced Thromboinflammation

**Figure 5:**
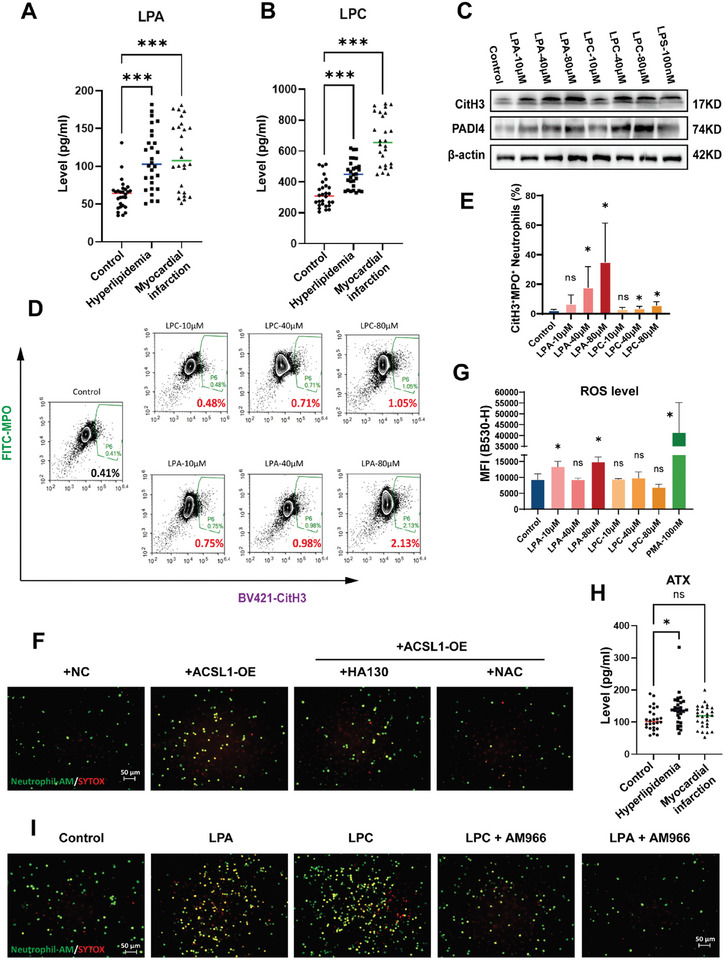
LPC/LPA derived from monocytes with ACSL1 overexpression promotes thromboinflammation by increasing NET release. A,B) LPA and LPC levels in the serum from the control, hyperlipidemia, and AMI group were measured using ELISA. (Control, *n* = 28; Hyperlipidemia, *n* = 28; Myocardial Infarction, *n* = 26; mean ± S.D; one‐way ANOVA). C) Western blot analysis of CitH3 and PADI4 in human neutrophils stimulated with 10/40/80 µm LPA or LPC, or 100 ng mL^−1^ LPS, at three hours post‐treatment. D,E) Representative flow cytometry graph for NETing neutrophils events [CitH3 + (Pacific blue) MPO + (FITC) double positive] and quantification analysis of NETing events ratio. Neutrophils were isolated from peripheral blood and processed with 10/40/80 µm LPA or LPC 3 h after treatment. (*n* = 6 each group; one‐way ANOVA). F) Fluorescently labeled neutrophils, treated with HA130 (an ATX inhibitor) and NAC (a ROS inhibitor), adhesion of fluorescently labeled neutrophils decreased. (*n* = 4 samples in each group; mean ± S.D). Scale bar: 50 µm. G) ROS levels of human neutrophils stimulated with 10/40/80 µm LPA or LPC, or 250 ng mL^−1^ PMA, at three hours post‐treatment were detected by DCFH probe using flow cytometry (*n* = 3 samples in each group; mean ± S.D). Scale bar: 100 µm. H) ATX levels in the serum from the control, hyperlipidemia, and AMI groups were measured using ELISA. (control, *n* = 28; H group, *n* = 28; A group, *n* = 26; mean ± S.D; one‐way ANOVA). I) After treatment with 80 µm LPA/LPC and AM966 (LPAR1 inhibitor), were applied to plates coated with endothelial cells and applied for 20 min at 75 rpm. (*n* = 4 samples in each group; mean ± S.D). ^*^ *p* < 0.05, ^***^ *p* < 0.001 versus control.

To validate that LPC and LPA levels regulate thromboinflamation, we first confirmed their increase in serum samples of hyperlipidemic and AMI patients (**Figure** **5**A,B). The abundance of LPC and LPA under hyperlipidemic states inferred that LPC was a pro‐atherosclerotic factor and LPA was associated with coronary heart disease, including AMI.^[^ ^15^ ^]^ Exogenous LPC/LPA resulted in elevated intracellular MPO and CitH3 levels in a concentration‐dependent manner (Figure 5C), which indicated increased activation of NETing neutrophils by these metabolites. LPA had a more pronounced pro‐NETing effect than LPC as lower concentrations could significantly raise the proportion of activated neutrophils (Figure 5D,E).

The activated neutrophils were also easily adhered to ECs with an increased dsDNA release (Figure 5F). LPC was reportedly an active lipoapoptosis‐inducing metabolite of saturated free FA, but we found that neither metabolite had a pro‐apoptotic effect on neutrophils even at high concentrations (80 µm) (Figure S9a,b, Supporting Information).^[^ ^16^ ^]^ LPC and LPA contribute to NETosis by enhancing oxidative stress (Figure 5G).

Elevated ATX in CM was also reflected in serum samples of hyperlipidemic patients (Figure 5H). The addition of LPAR1 and ATX inhibitor (AM966 and HA130) as well as ROS inhibitor (NAC) alleviated the adhesion of CM/LPA/LPC treated neutrophils to ECs (Figure 5F,I). This solidifies the role of the LPC‐ATX‐LPA axis for ACSL1‐overexpressed monocytes in promoting NETosis and subsequently thromboinflammation.

### 2.6 ACSL1 Knockdown Mitigates Thromboinflammation

**Figure 6:**
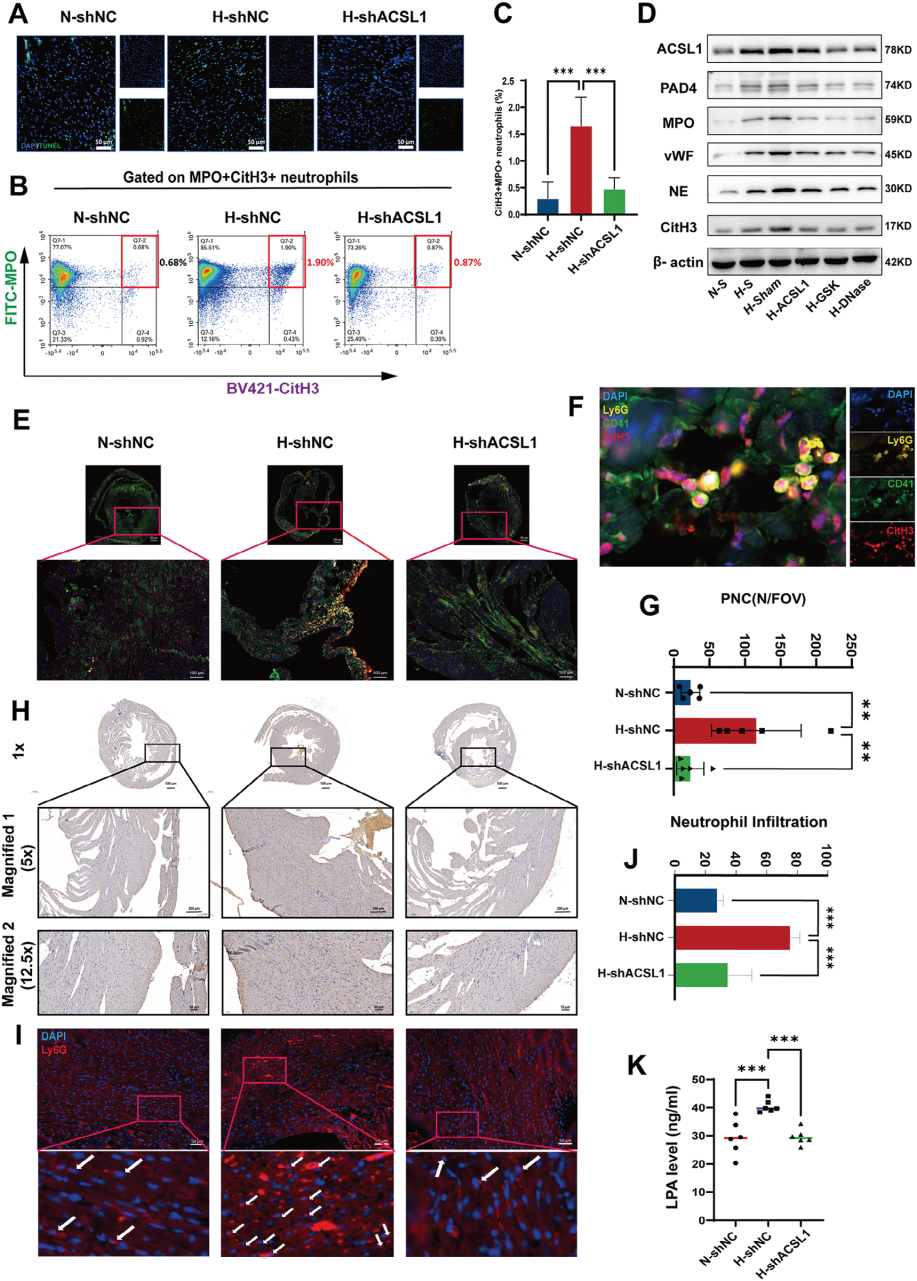
ACSL1 knockdown ameliorated thromboinflammation and myocardial IR Injury in ApoE^−/−^ mice. A) Representative images of apoptotic cells from one mouse of each group. Scale bar: 50 µm. B) Representative flow cytometry density plots using fresh blood gated for NETing neutrophils. C) Quantification of CitH3^+^MPO^+^ neutrophils by flow cytometry. (*n* = 6 each group; one‐way ANOVA). D) Western blot analysis of proteins in the infarct border zone tissue of mouse hearts. The blot shows the expression levels of ACSL1 (78 kDa), PADI4 (74 kDa), MPO (59 kDa), vWF (45 kDa), NE (30 kDa), CitH3 (17 kDa), and β‐actin (42 kDa) across different experimental groups. E–G) Immunofluorescence staining and quantification of platelet‐neutrophil complexes (PNCs) in the infarct border zone. (E) and (F) show PNCs marked by CD41 (red), Ly6G (yellow), and CitH3 (green) across different groups. Insets highlight marker localization differences. The rightmost panel (F) is a high‐magnification image of the apoE^−/−^ (HCD) + pAAV‐shNC group, displaying individual channels for DAPI (blue), Ly6G (yellow), CD41 (red), and CitH3 (green). (G) quantifies the number of PNCs per field of view (N/FOV), showing significant increases in the HCD group compared to controls. (*n* = 10 each group; one‐way ANOVA). H) IHC staining of ACSL1 in heart sections from ApoE^−/−^ (NCD) + shNC, ApoE^−/−^ (HCD) + shNC and ApoE^−/−^ (HCD) + shACSL1. Scale bar: 200 µm. I,J) Immunofluorescence staining was performed on the hearts of mice, neutrophils (red Ly6B.2‐positive cells as indicated by white arrow) infiltrated in the heart tissue of control, hyperlipidemia with or without AAV9‐shACSL1 injected. (*n* = 5 in each group; mean ± S.D; one‐way ANOVA). Scale bar: 50 µm. K) Plasma LPA levels were quantified by ELISA in ApoE^−/−^ (NCD) + shNC group and ApoE^−/−^ (NCD) + shACSL1 group. (*n* = 5 in each group; mean±S.D;. ^**^ *p* < 0.01, ^***^ *p* < 0.001 versus ApoE^−/−^ (NCD) + shNC.

We employed an adeno‐associated virus (AAV) vector to knock down ACSL1 in male HCD mice in vivo. Previous research has demonstrated that ACSL1 knockdown leads to a reduction in ATX levels.^[^ ^15a^ ^]^ LPC can be hydrolyzed by ATX to produce LPA, a bioactive lipid involved in various physiological and pathological processes.^[^ ^15b^ ^–^ ^17^ ^]^Consistent with these findings, our study observed a significant decrease in plasma LPA concentrations in the HCD + shACSL1 group compared to the HCD group. This reduction in LPA levels was associated with less severe mIRI, as indicated by reduced apoptotic cell presence and TUNEL staining (**Figure** **6**A,K). Cardiac ACSL1 expression was significantly upregulated in the peri‐infarct regions of HCD mice, as evidenced by Western blot analysis (Figure 6D). This prompted the investigation of ACSL1 as a potential therapeutic target for ameliorating mIRI.

Flow cytometry analysis of peripheral blood samples revealed a marked reduction in NETosis activity in the shACSL1 group compared to the HCD group (Figure 6B,C). This decrease in NETosis was accompanied by lower expression levels of adhesion, inflammation, and thrombosis‐related molecules in the shACSL1 group, suggesting that ACSL1 knockdown mitigates the heightened thromboinflammation response associated with severe dysregulated lipid metabolism (Figure 6D). Injection of AAV9‐shACSL1 achieved a comparable mitigating effect on these markers, further supporting the therapeutic potential of targeting ACSL1.

Immunofluorescence staining of the infarct border zones in mouse hearts demonstrated a significant decrease in PNCs in the shACSL1 group compared to the HCD group (Figure 6E–G). This finding highlights the pivotal role of ACSL1 in mediating the formation of PNCs and the ensuing thromboinflammation response in mIRI.

Moreover, immunohistochemistry (IHC) and immunofluorescence staining of heart tissues corroborated these findings by showing a substantial reduction in neutrophil infiltration and thromboinflammation markers in the shACSL1‐treated group (Figure 6H–J). The analysis of plasma LPA levels by ELISA further supported these results, revealing a significant decrease in the HCD + shACSL1 group compared to the HCD group, thereby underscoring the role of ACSL1 in lipid metabolism and its potential as a therapeutic target for mitigating thromboinflammation (Figure 6K).

These analyses confirm the crucial role of ACSL1 in the pathogenesis of mIRI and suggest that targeting ACSL1 can effectively reduce thromboinflammatory processes in hyperlipidemic conditions.

## 3 Discussion

mIRI is a comparatively more severe event in hyperlipidemic patients. Previous studies identified ROS as key factors that lead to oxidative stress injury in patients with hyperlipidemia, thereby damaging the myocardium.^[^ ^18^ ^]^ ACSL1 exerts its function by directing the metabolic partitioning of FAs toward β‐oxidation in adipocytes, and its overexpression reduces FA oxidation through the PPARγ pathway.^[^ ^19^ ^]^ Through bulk RNA‐seq, we revealed ACSL1 as a key mediator between hyperlipidemia and AMI. Metabolomics study identified ACSL1 as a critical promotor of NET formation under hyperlipidemic conditions. Finally, our study pinpointed LPC and LPA as key downstream metabolites and established the ACSL1‐LPC‐ATX‐LPA axis as an intricate metabolic pathway that aggravates thromboinflammation in AMI due to hyperlipidemia.

The interplay between inflammation and plasma coagulation is a complex network with disrupted molecular and cellular interactions.^[^ ^20^ ^]^ Although monocytes influence neutrophil function, including tissue factor (TF) expression, NETosis, and ROS‐induced apoptosis to initiate coagulation, the full scope regarding monocyte‐neutrophil interaction in inflammation is yet fully clarified.^[^ ^21^ ^]^ When trained with oxLDL and stimulated with LPS, human monocytes exhibited characteristics of inflammasome‐mediated trained immunity, which could explain their role in enhancing NETosis when co‐cultured with neutrophils.^[^ ^22^ ^]^

The ACSL1 gene is a NETosis‐related gene in chronic inflammation or autoimmune diseases.^[^ ^23^ ^]^ Despite THP1 monocyte cell lines stably overexpressing ACSL1 exhibiting a pronounced tendency toward osteoclast differentiation (Figure S10a, Supporting Information), we observed that oxLDL at high concentrations (100 µg mL^−1^), while markedly promoting ACSL1 overexpression, did not significantly increase their inflammatory phenotype (Figure S10b–e, Supporting Information), suggesting an alternative mechanism by which they might promote NET formation. Our research shows that in addition to producing neutrophil chemokines (e.g., CXCL2), hyperlipidemia‐induced metabolic reprogramming in monocytes enhances NETs release through regulatory metabolites, specifically LPC and LPA.^[^ ^24^ ^]^

LPC and LPA are complex metabolites with an array of receptors and are involved in various cellular functions, including platelet aggregation, cell proliferation and migration, and smooth muscle cell contraction.^[^ ^25^ ^]^ The functions of LPC and LPA are more intricate than previously assumed: prior research has identified contrasting roles in cell proliferation, autophagy, and apoptotic phenotypes.^[^ ^13^, ^26^ ^]^ Regarding their impact on ROS accumulation, current research presents a conflicting conclusion. As a component of oxLDL, LPC can activate NADPH oxidase and ROS. However, we did not find the anticipated high levels of ROS in neutrophils stimulated with LPC. Intriguingly, higher concentrations of LPC appeared to further inhibit ROS accumulation in neutrophils, aligning with the apoptotic phenotype observed in neutrophils under LPC stimulation. Although these findings suggest the protective role of LPC on neutrophils, the capacity of LPC to promote NET formation is concentration‐dependent, indicating that this promotion does not rely on ROS accumulation. Instead, the facilitation of calcium translocation into cells may play a more significant role. Conversely, LPA demonstrates opposite functions related to ROS accumulation across different studies.^[^ ^27^ ^]^

LPA surpasses LPC in elevated ROS levels in neutrophils, giving it a stronger capacity to promote NETosis, which may also be its primary mechanism of action. LPA is reported to possess a stronger ability to promote calcium influx than LPC, which can be another crucial mechanism behind its induction of NET formation.^[^ ^28^ ^]^ Altogether, ACSL1 overexpression under hyperlipidemic conditions accelerates the LPC‐ATX‐LPA axis, thereby promoting the increase of ROS level and calcium influx, and finally increasing NET formation.

NETosis facilitates the delivery of von Willebrand factor to the endothelial plasma membrane, where it subsequently binds to platelets via the glycoprotein Ibα receptor, a critical step in thrombus formation.^[^ ^29^ ^]^ Our findings indicate that CM from monocytes overexpressing ACSL1 enhances the adhesion of neutrophils to endothelial cells. Notably, this adhesion was significantly reduced following treatment with AM966, an LPAR1 inhibitor, suggesting that LPA, derived from the CM of ACSL1‐overexpressed monocytes, is a meaningful effector. This identifies a potential therapeutic target.

However, there are some limitations to this study. We showed that the myocardial infarction area was notably reduced by interfering with DNase I and GSK484 treatment, knockdown ACSL1 in vivo also reduced the myocardial infarction area in mIRI mice. However, the direct evidence that demonstrates the ACSL1‐mediated thromboinflammation effect is limited. Further investigations could be useful to address issues such as cardiac function or cardiac structure in ACSL1 overexpressed mIRI mouse models with DNase I and GSK484 treatment in vivo. More comprehensive experimental evidence needs to be supplemented, such as Evans blue/TTC staining is suitable to show the infarct area and area at risk (AAR) after reperfusion.

By combining and extending current knowledge regarding hyperlipidemia and its risk in AMI and aggravating mIRI, our study further provides mechanistic insight into how the hyperlipidemia state enhances the thrombotic and inflammation process through monocyte‐secreted lipid metabolites that regulate pathologic, chronic neutrophil activation. Furthermore, we uniquely identified the role of the LPC‐ATX‐LPA axis in mIRI thromboinflammation during hyperlipidemic conditions, and LPC/LPA serves as key metabolites that are attractive therapeutic targets in the future.

## 4 Experimental Section

### Patients and Specimens

The study was reviewed and approved by the ethics committee of Shanghai Pudong Hospital, Fudan University Pudong Medical Center (2023‐QWJWRC‐R‐02). Signed written informed consent was obtained from the patient. Blood samples were collected from 99 patients, among them 7 hyperlipidemic patients and 10 healthy controls were enrolled for bulk RNA‐seq. 27 AMI patients, 28 hyperlipidemic patients, and 28 healthy adults were enrolled for validation. Detailed demographics were obtained from hospital records and summarized in Table S1 (Supporting Information). At least 15 mL of blood was collected in heparin‐coated tubes. Five ml of blood were centrifuged at 8000 rpm for 10 min to separate and collect the upper plasma layer, which was stored at −80 °C.

### Bulk and Single‐Cell Sequencing Analysis

For control and hyperlipidemic patients RNA‐sequencing, fresh blood samples were collected in heparin sodium anticoagulated tubes. PBMCs were isolated using the standard Ficoll‐Paque density‐gradient method according to the manufacturer's instructions. The top plasma layer was collected for other measurements. The total RNA extracted from PBMC pellet was used to prepare sequencing libraries using a TruSeq RNA Prep Kit (Illumina, San Diego, CA, USA), and sequencing was used by a genome analyzer HiSeq X Ten (Illumina). For public bulk RNA‐seq, the datasets GSE48060 and GSE66360 were obtained from NCBI.

Briefly, the raw data were processed with Kallisto (Near‐optimal probabilistic RNA‐seq quantification). Then differential expression analysis and correlation analysis, enrichment analysis, immunocyte infiltration analysis, consensus clustering analysis, validation of clustering and immune‐related analysis, three machine learning methods (Random Forest, SVM‐RFE, and LASSO), gene set enrichment analysis, and validation of the model genes was performed following standard analysis.

For single‐cell sequencing analysis, the datasets were obtained from NCBI (https://www.ncbi.nlm.nih.gov/geo/query/acc.cgi). The data contained mixed samples of blood and heart from AMI mouse models.^[^ ^30^ ^]^ Briefly, the matrix expression and meta information were first obtained from the data. According to their annotation, the cells were divided into 8 immune cell clusters excluding the doublets clusters. Following that, the expression of *Acsl1* in monocytes were calculated. The result was presented by Vlnplot (Seurat V3) and the p‐value was conducted by ggpubr package.

### Non‐Targeted Lipid Metabolome Detection and Analysis

For untargeted lipidomics based on LC‐MS, sample preparation is conducted according to the following process:

In LC‐MS lipidomics, begin by transferring the cell‐containing culture medium to 15 mL Corning tubes (ensuring a cell count ≥ 1 × 10^6^). Centrifuge at 750 × g for 5 minutes at 4 °C, wash with PBS, and aspirate the solution. Next, introduce 200 µL H2O and 1.5 mL methanol, vortex the mixture, and transfer it to a 15 mL glass tube. Following this, incorporate 5 mL MTBE, vortex for 1 min, and shake for 1 h at room temperature. Supplement 1.25 mL H2O, vortex again, and perform centrifugation at 1000 × g for 10 min at 4 °C. Carefully transfer the upper liquid phase to 1.5 mL tubes. Concentrate the samples without applying heat, store them at −80 °C, and ensure transportation on dry ice. (All reagents used in this process are of HPLC grade.)

The LC/MS system was used for metabolomic analysis. Positive ion mode: 0.1% formic acid aqueous solution and 0.1% formic acid acetonitrile. Negative ion mode: 0.1% formic acid aqueous solution and 0.1% formic acid acetonitrile. The raw data were collected using thermo orbitrap Exploris480 and then processed by Progenesis QI software based on the Progenesis QI software online METLIN database and Biomark's self‐built library for identification, and at the same time, theoretical fragment identification and mass deviation were all within 100 ppm.

LC‐MS raw data files (.raw) were converted to mzXML format using ProteoWizard (version 3.0.20315) 65. El‐MAVEN (version 0.7.0 or 0.12.0) was used to generate a peak table containing m/z, retention time, and intensity for peaks. Parameters for peak picking were the defaults except for the following: mass domain resolution is 10 ppm; time domain resolution is 15 scans; minimum intensity is 1000; minimum peak width is 5 scans. The resulting peak table was exported to a.csv file. Redundant peak entries due to imperfect peak picking process are removed if two peaks are within 0.1 min and their m/z difference is within 2 ppm. Background peaks are removed if the intensity in the procedure blank sample is > 0.5‐fold of that in biological samples.

For lipid validation, LysoPC (Lysophosphatidylcholine) and PE (Phosphatidylethanolamine) levels in the THP1 monocytes were measured by assay Kit (Colorimetric/Fluorometric) following the guidelines provided by manufacturers (Abcam ab273332; Sigma MAK361‐1KT).

### Cell Culture

Primary neutrophils were isolated from fresh blood. THP1 and primary human umbilical vein endothelial cells (HUVEC) were obtained from ATCC. Primary neutrophils were cultured and cultured in a completed culture medium with RPMI 1640 medium supplemented with 10% fetal bovine serum, 100 units mL^−1^ penicillin/streptomycin at 37 °C in a 5% CO_2_ incubator. HUVEC was cultured in Endothelial Cell Medium (ScienCell, USA). All antibodies and main reagents used in cell assay are listed in Table S2 (Supporting Information).

### Cell Adhesion Assay

ECs were treated in 12‐well plates and incubated overnight. Neutrophils were collected at 1200 rpm for 3 min at room temperature. The cells were counted and adjusted to a concentration of 1 × 10^6^ cells mL^−1^ in a medium supplemented with 10% FBS. Once the ECs had been incubated, they were rinsed with 1 mL of RPMI 1640 + 10% FBS at room temperature. After being pre‐treated with SYTOX Red (Thermo, S34859) and Calcein AM (yeasen 40719ES60), 1 × 10^6^ neutrophils were added per mL per dish in the same medium. The setup was incubated at room temperature for 20 min at 75 rpm then gently washed with with 1 mL of PBS at room temperature. One ml of 1% PFA in PBS was added and the cells were allowed to fix for 5 min at room temperature. After fixing, the PFA was removed. Finally, 3 pictures of each dish were taken immediately to ensure that the cells were properly prepared and imaged for accurate analysis.

### Cell Counting Kit‐8 Assay

Cell viability was analyzed using the CCK‐8 assay. Cells were seeded in 96‐well plates at 10 000 cells per well. Before testing, the cells were incubated with 10 µL CCK‐8 solution for 2 h at 37 °C. The absorbance was measured at 450 nm by using a full wavelength microplate reader. All experiments were performed in triplicate.

### ACSL1 Stable Overexpression/Knockdown Cell Line Establishment

A lentivirus carrying the ACSL1(NM 001995.5) gene was constructed in pCDH‐CMV‐MCS‐EF1‐Puro and pSLenti‐U6‐shRNA‐CMV‐EGFP‐F2A‐Puro‐WPRE (Targeting sequence: ATCATAGTTGTCATGGATGCC) vector by OBiO Technology Co. LTD (Shanghai, China). THP1 monocytes were seeded in a 12‐well plate and then infected with the lentivirus according to protocols as recommended by the manufacturer. After 24 h, the medium was replaced with a complete medium. To obtain a stable ACSL1 overexpressing cell line, the lentivirus‐infected cells were selected by incubation with 2 µg mL^−1^ of puromycin. The expression of ACSL1 in THP1 cell lines stably infected with a lentivirus was examined by Western blot and RT‐PCR.

### Immunocytofluorescence

First, 1 × 10^6^ neutrophils were washed with PBS and polylysine‐coated on slides using a cytospin technique at 600 rpm for 3 min. The slides were fixed in 4% formaldehyde and washed three times with cold PBS. Cells were permeabilized with 0.1% Triton X‐100, washed three times in cold PBS, blocked in 1% BSA for 1 h, and incubated overnight with primary antibodies (1:200 rabbit anti‐CitH3 polyclonal antibody;1:100 rat anti‐MPO monoclonal antibody) at room temperature. Cells were washed three times in PBS with 0.05% Tween and incubated with secondary Ab (Alexa Fluor 488 conjugated) for 1 h. After three washes with PBS, cells were counterstained and mounted using VECTASHIELD HardSet Antifade Mounting Medium with DAPI (catalog number H‐1500; Vector Laboratories). Confocal images were collected using a Plan‐Apochromat 63×/1.40 oil DIC M27 objective lens (Zeiss Observer 7; Gottingen, Germany) with excitation via a 590‐nm diode‐pumped solid‐state laser and a 405‐nm line of an argon ion laser, and the optimized emission detection bandwidths were configured using Zeiss ZEN pro software.

### Neutrophil Isolation and NETosis Activation

Isolation of neutrophils was performed with the use of peripheral blood neutrophil isolation kid (Solarbio, Beijing, China) according to the manufacturer's instructions. Neutrophils were suspended in RPMI‐1640 media (GIBCO, Grand Island, NY, USA) with 3% fetal bovine serum (Fisher Scientific, PGH, USA) at a concentration of 5 × 10^6^ cells mL^−1^. Immediately after isolation, neutrophils were incubated at 37 °C, 5% CO_2_, 4 h in 100 mm tissue culture‐treated Petri dishes (Fisher Scientific) with 250 ng mL^−1^ Phorbol 12‐myristate 13‐acetate (Sigma–Aldrich, Burlington, MA, USA) to stimulate NETosis.

### Animals and mIRI Model

ApoE^−/−^ adult (18–20 weeks old; male) mice in C57/bl6 background were purchased from Nanjing JKbiot and housed in a specific pathogen‐free facility at the Fudan University Pudong Medical Center (Shanghai, China). A group of mice was fed with HCD, and the control group was fed with a normal diet. For selected experiments, the HCD mice were treated with or without GSK484 (4 mg kg^−1^, IP, MCE, USA), DNase I (10 mg kg^−1^, IP, Solarbio, China), or vehicle (10% DMSO in PBS, IP) for 5 days. The ApoE^−/−^ HCD+shACSL1 (NM_001302163.2) group was administered with the AAV9‐miRNA‐shACSL1 and AAV9‐miRNA‐shNC by tail vein injection. Each group consisted of 6 mice.

A mouse model involving mIRI was used, as described previously. Briefly, mice were placed in a stereotaxic frame following anesthesia with isoflurane (2%). Left‐sided thoracotomy was performed and the left anterior descending (LAD) artery was temporarily ligated for 30–60 min, followed by reperfusion for 24 h (acute). Sham‐operated mice underwent the same procedure without LAD artery ligation.

All animals were treated and cared for under the National Institutes of Health Guide for the Care and Use of Laboratory Animals (Revised, 1996), and all protocols were approved by the Fudan University Pudong Medical Center Institutional Animal Care and Use Committee (ref: 2023‐D‐QWJWRC‐04).

### TUNEL Assay

Apoptotic cells in tumor tissues were analyzed by terminal deoxynucleotidyl transferase‐mediated UTP nick end labeling (TUNEL) staining. Apoptotic cell nuclei were stained with green fluorescence according to the manufacturer's (TUNEL FITC Apoptosis Detection Kit, Vazyme, Jiangsu, China) protocol. Cell nuclei were stained with DAPI.

After the heart tissues were washed with precoded saline 3 times and wiped dry, the heart was immediately frozen in a refrigerator at −20 °C for 20 min and then cut into 3–5 mm slices. The slices were incubated at 37 °C in a 2% solution of TTC (Solarbio, China) (dissolved in phosphate buffer saline, pH 7.4) for 30 min. The infarction size of heart tissue was analyzed by ImageJ software (National Institutes of Health, USA).

### Immunofluorescence

Mice were sacrificed after completion of mIRI at different time points and myocardial tissues were harvested. Hearts were isolated, fixed in 4% paraformaldehyde overnight at 4 °C, then embedded in paraffin after dehydration. Sectioning and wheat germ agglutinin staining was performed as previously described.^[^ ^31^ ^]^ Briefly, cardiac sections were blocked using 10% normal serum followed by staining with Alexa Fluor‐594 conjugated WGA antibody (ThermoFisher Scientific) for 1 h at room temperature. All slides were mounted and analyzed with the EVOS FL cell imaging system. For ACSL1 and Ly6B.2 immunostaining to label, hearts were fixed with 0.5% PFA overnight at 4 °C and embedded with OCT after dehydration. Cardiac sections were washed twice with 0.1% PBS‐Tween for 5 min and permeabilized with 0.2% PBS‐Triton for 15 min at RT. The sections were then blocked with 5% BSA for 1 h at RT. After that, the sections were incubated overnight at 4 °C with primary antibody against Ly6B.2, Ly6G (Cell signaling technology, 68590S), CD41 (ABclonal, A11490, 1:100) and CitH3 (Abcam, ab281584, 1:100) and fluorescent secondary antibody (1:500, ThermoFisher Scientific) and Alexa Fluor‐488 conjugated WGA antibody (ThermoFisher Scientific) for an additional 1 h at RT. Immunofluorescence staining was utilized to confirm neutrophil infiltration and CitH3 expression in the tissue surrounding myocardial infarction. The control group and GSK484/DNase I‐treated mice heart tissues were fixed with 4% paraformaldehyde for 15 min. Then the cells with 0.3% Triton X‐100 for 5 min and blocked with 3% BSA in PBS.

### Western Blot Analysis

Following different treatments, THP1 and neutrophils were harvested and incubated for 30 min with lysis buffer (10× Lysis Buffer, Cell Signaling Technology Inc., Danvers, MA, USA). The protein lysates were prepared and resolved using 12% SDS‐PAGE. Cellular proteins were transferred to Immuno‐Blot PVDF membranes (Millipore, USA) by electro‐blotting. The membranes were then blocked with 5% non‐fat milk in TBST for 2 h followed by incubation with primary antibodies against p‐STAT3 and STAT3, ACSL1, NE, PADI4, and CitH3 at a 1:1000 dilution at 4 °C overnight. The blots were then washed three times with TBST and incubated for 2 h with HRP‐conjugated secondary antibody (CST, USA). Immunoreactive bands were developed using an Amersham ECL Plus Western Blotting Detection System (Millipore, USA) and visualized with an iBright CL750 imaging system (Thermo, USA).

### RT‐PCR and Primer Sequences

Total cellular RNA was extracted using the Trizol Reagent (Takara, Japan) following the manufacturer's instructions. Complementary DNA (cDNA) was synthesized using 1 µg of total RNA following the guidelines from the high‐capacity cDNA reverse transcription kit with gDNA remover (Takara, Japan). RT‐PCR was performed in triplicate using the BrightCycle Universal SYBR Green qPCR Mix with UDG (Abclonal, China) with specific primers (Table S3, Supporting Information) and a Roche LightCycler 480 II RT‐PCR system.

### Flow Cytometry

For apoptosis assay, primary neutrophils were resuspended in 500 µL binding buffer, and collected by centrifugation at 1200 rpm for 5 min. The cell suspension was stained with 5 µL Annexin V‐FITC and 5 µL PI in darkness for 10 min at 25 °C. Apoptotic cells were detected by flow cytometer (NovoCyte D3000, Agilent, California, USA). For the detection of total ROS, primary neutrophils were collected and the diluted CM‐H2DCFDA fluorescent probe (D6470, Solarbio, China) was added to the cells for 20 min incubation at 37 °C. After cells were washed with serum‐free medium 3 times and resuspended in 50 µL PBS, a flow cytometer was used to detect the level of ROS.

Citrated blood freshly collected from AMI patients, hyperlipidemia patients, and healthy donors was fixed with 4% paraformaldehyde for 10 min at room temperature. Cells were washed with FACS buffer and stained with surface staining antibodies including anti‐CD45 (APC/CY7, BioLegend 304054), anti‐CD16 (PE, BioLegend 302007), anti‐CD66b (APC, BioLegend 305117) for 30 min on ice in the dark. Then the cells were treated with Permeabilization Wash Buffer (Yeasen, China) for 30 min at room temperature in the dark. Anti‐citrullinated histone H3 (ab281584) and anti‐MPO (PE, BD 341642) antibodies were incubated for another 30 min, followed by donkey anti‐rabbit lgG (Brilliant Violet 421m, BioLegend 406410) antibodies incubation. Samples and analyzed by flow cytometry (NovoCyte D3000, Agilent, California, USA). For fresh peripheral blood from mice, the following flow cytometry antibodies were used for staining: anti‐Ly‐6G (PE/Cyanine7, BioLegend 127617), anti‐CD16 (PerCP, BioLegend 101230), and anti‐CD45 (APC, BioLegend 103112). The raw data were analyzed by Flowjo V10.

### Statistical Analysis

For the processing of data, qRT‐PCR and quantitative data of Western blot were normalized to 1 in the control group, and other data were presented as primary measured values. All the data were shown as means± S.D. The numbers of sample sizes for each experiment were indicated in the figure legend. GraphPad Prism 8.0 (GraphPad Software, Inc., USA) was applied for statistical analysis. Data between 2 groups were assessed using Student's t‐test. Data in multiple groups were evaluated using analysis of variance (ANOVA). All tests were two‐tailed. A p‐value < 0.05 was considered to be statistically significant. ^*^ *p* < 0.05, ^**^ *p* < 0.01, ^***^ *p* < 0.001, and *p* < 0.05 mean no significance.

## Conflict of Interest

The authors declare no conflict of interest.

## Author Contributions

S.J. and J.T. contributed to the whole project conception and design. The manuscript was prepared and revised by S.J., and X.L. The main clinical and cell experiments were performed by X.L., B.C., and S.Y. The main animal experiments were completed by B.C. Data analysis was performed by J.L, Q.S. Clinical samples were processed by G.C., K.K., Y.D. J.W., and Y.L. Founding support was given by J.D., B.Y., and S.J. All authors read and approved the final manuscript.

## Supporting information



Supporting Information

## Data Availability

The data that support the findings of this study are available from the corresponding author upon reasonable request.
