# Supplementary material for: ACSL1 Aggravates Thromboinflammation by LPC/LPA Metabolic Axis in Hyperlipidemia Associated Myocardial Ischemia‐Reperfusion Injury
Source: Adv Sci (Weinh). 2025 Jan 23;12(11):2406359. doi: 10.1002/advs.202406359 (PMC11923997; doi:10.1002/advs.202406359)
Supplement: Supplementary file 1 — Supporting Information [file ADVS-12-2406359-s001.pdf]

ADVANCED SCIENCE
Open Access

Supporting Information

for *Adv. Sci.*, DOI 10.1002/advs.202406359

ACSL1 Aggravates Thromboinflammation by LPC/LPA Metabolic Axis in Hyperlipidemia Associated Myocardial Ischemia-Reperfusion Injury

Shuai Jiang, Xueguang Lin, Bo Chen, Gang Chen, Kristine J.S. Kwan, Jing Liu, Qi Sun, Jie Wang, Yijie Lu, Jindong Tong, Ying Deng, Bo Yu* and Jingdong Tang**

Supplementary information

Hyperlipidemia Mediated ACSL1-LPC-LPA Axis Amplifies Myocardial Ischemia-Reperfusion Injury by Promoting Thromboinflammation

Shuai Jiang[#1], Xueguang Lin[#1], Bo Chen[1], Gang Chen[2], Kristine J.S. Kwan[1], Jing Liu[3], Qi Sun[4], Jie Wang[1], Yijie Lu[1], Jindong Tong[1], Ying Deng[1], Bo Yu*[1,5], Jingdong Tang*[1].*

[1] Shanghai Key Laboratory of Vascular Lesions and Remodeling, Department of Vascular Surgery, Shanghai Pudong Hospital, Fudan University Pudong Medical Center, Shanghai, 201399, China
[2] Department of Cardiology, Shanghai Pudong Hospital, Fudan University Pudong Medical Center, Shanghai, 201399, China
[3] State Key Laboratory of Genetic Engineering, Collaborative Innovation Center for Genetics and Development, School of Life Sciences, and Human Phenome Institute, Fudan University, Shanghai, 200438, China
[4] Department of Endocrinology and Metabolism, Affiliated Hospital of Nantong University, Nantong, 226006, China
[5] Department of Vascular Surgery, Huashan Hospital, Fudan University, Shanghai, 200040, China
*Corresponding author.
E-mail: ydeng14@fudan.edu.cn; yubo120@hotmail.com; drtangjingdong@fudan.edu.cn;

Figure. S1

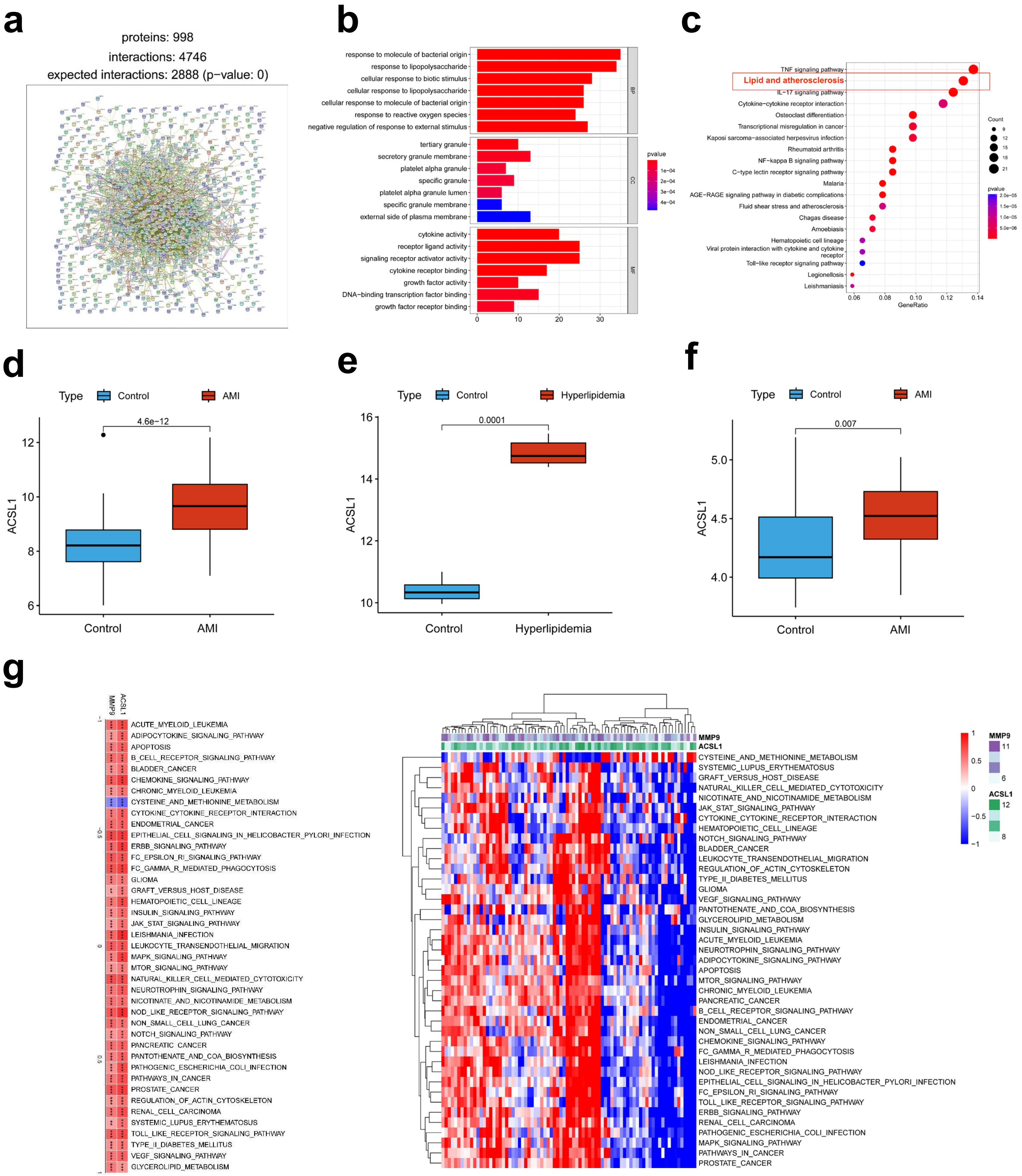

a. Protein-protein interaction (PPI) network analysis. The edges are colored to denote experimentally confirmed interactions and/or associations in curated databases (blue edges), and other sources of evidence (gray edges). **b, c.** GO and KEGG functional enrichment analysis. **d-f.** Validation of ACSL1 and MMP9 in external and internal cohorts (The data showed as means ± S.D; t-test). **g.** Heatmaps illustrating GSVA-based expression levels and pathway association scores for ACSL1 and MMP9 across various biological pathways, where red indicates higher activity or positive correlation, and blue indicates lower activity or negative correlation.

Figure. S2

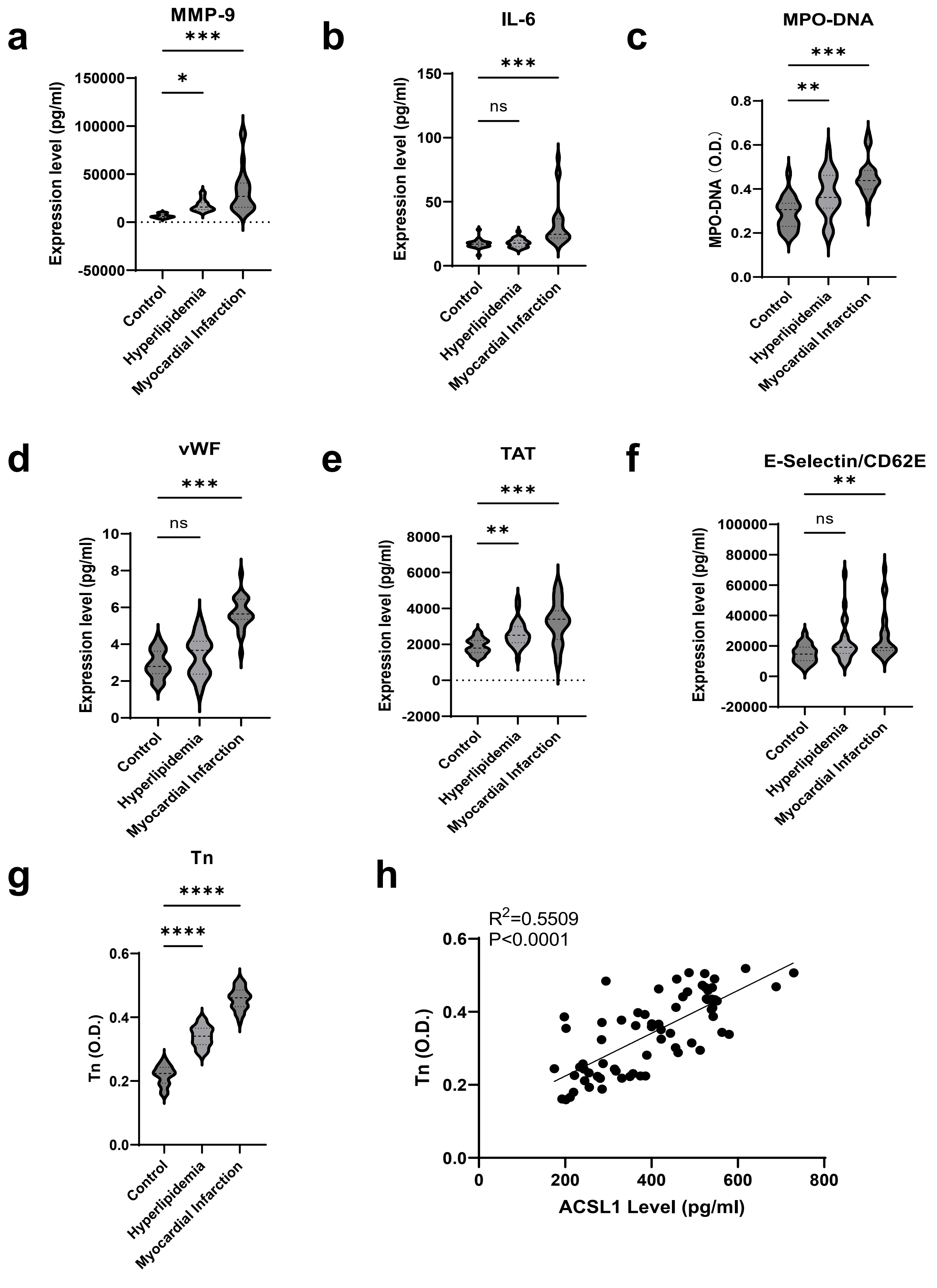

a-g. MMP9, IL-6, MPO-DNA complex, vWF, E-Selectin/CD62E, Tn were measured by multiple luminex assay and ELISA. h, Correlation analysis between Tn and ACSL1 level. (N group: n = 28; H group: n = 28; A group: n=26; one-way ANOVA. The data showed as means ± S.D; one-way ANOVA; *P < 0.05, **P < 0.01, ****P < 0.0001 vs. N group)

Figure. S3

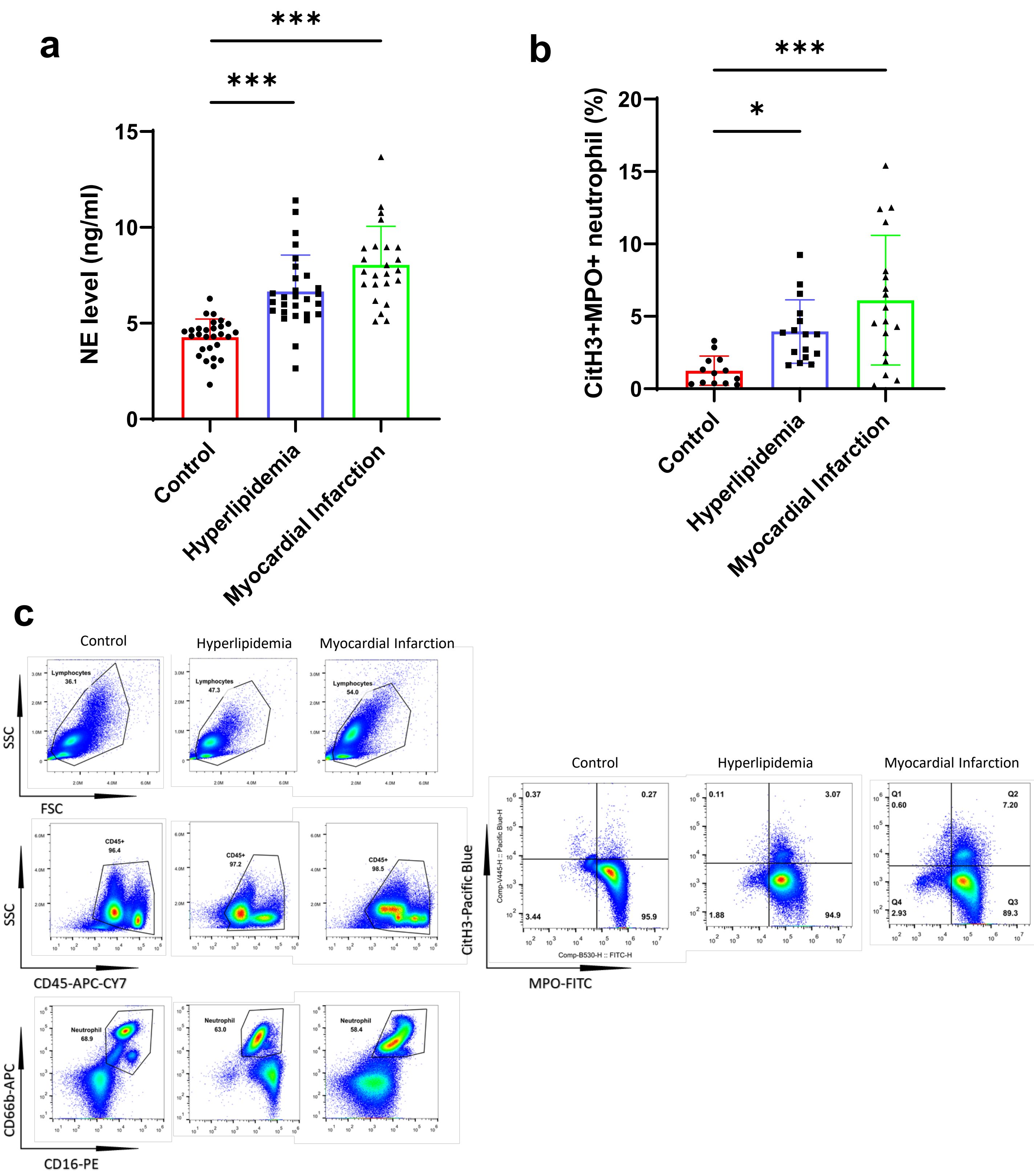

a. Serum elastase levels detected by ELISA showed significantly higher in AMI (n=26) and hyperlipidemia patients (n=26) when compared to healthy volunteers (n=28). **b.** Percentage of CitH3^{+}MPO^{+} double-positive cells across three distinct groups by flow cytometry analysis. **c.** Gating strategy to identify neutrophils in blood. Data were presented as mean ± SEM and analyzed by one-way ANOVA test. *P < 0.05, **P < 0.01, ***P < 0.001, ****P < 0.0001, NS: no significance.

Figure. S4

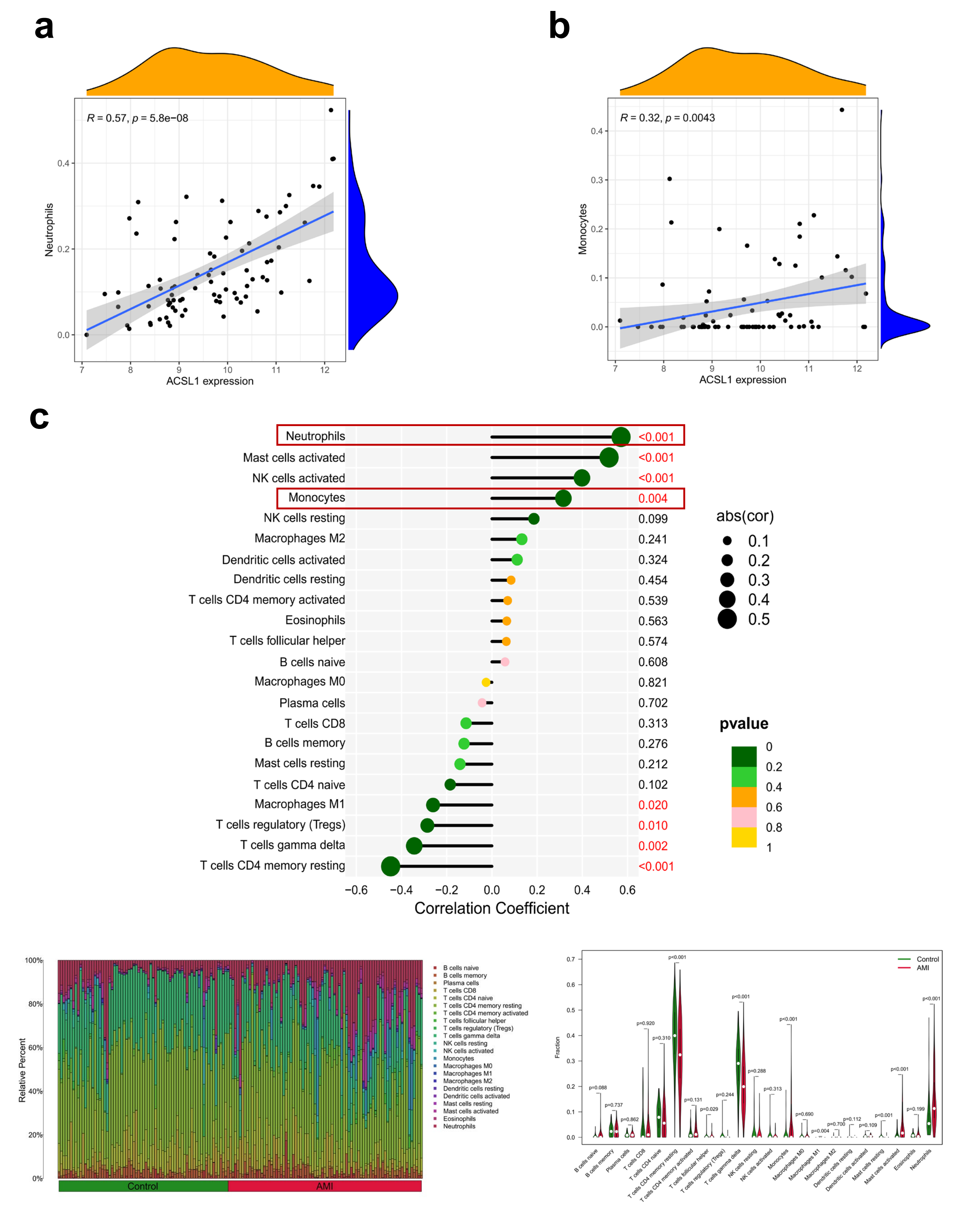

Immune cell infiltration landscape. **a-b.** Correlation analysis of ACSL1 expression and infiltration immune cells. **c.** (upper panel) The relationship between IFI27and immune cell infiltration level. (lower left panel) The boxplot diagram indicates the relative percentage of different types of immune cellinfiltration between AMI patients and healthy controls. (lower right panel) The violin graph shows the difference in immune infiltration between the two groups. *P < 0.05, **P < 0.01, ***P < 0.001, ****P < 0.0001, NS: no significance.

Figure. S5

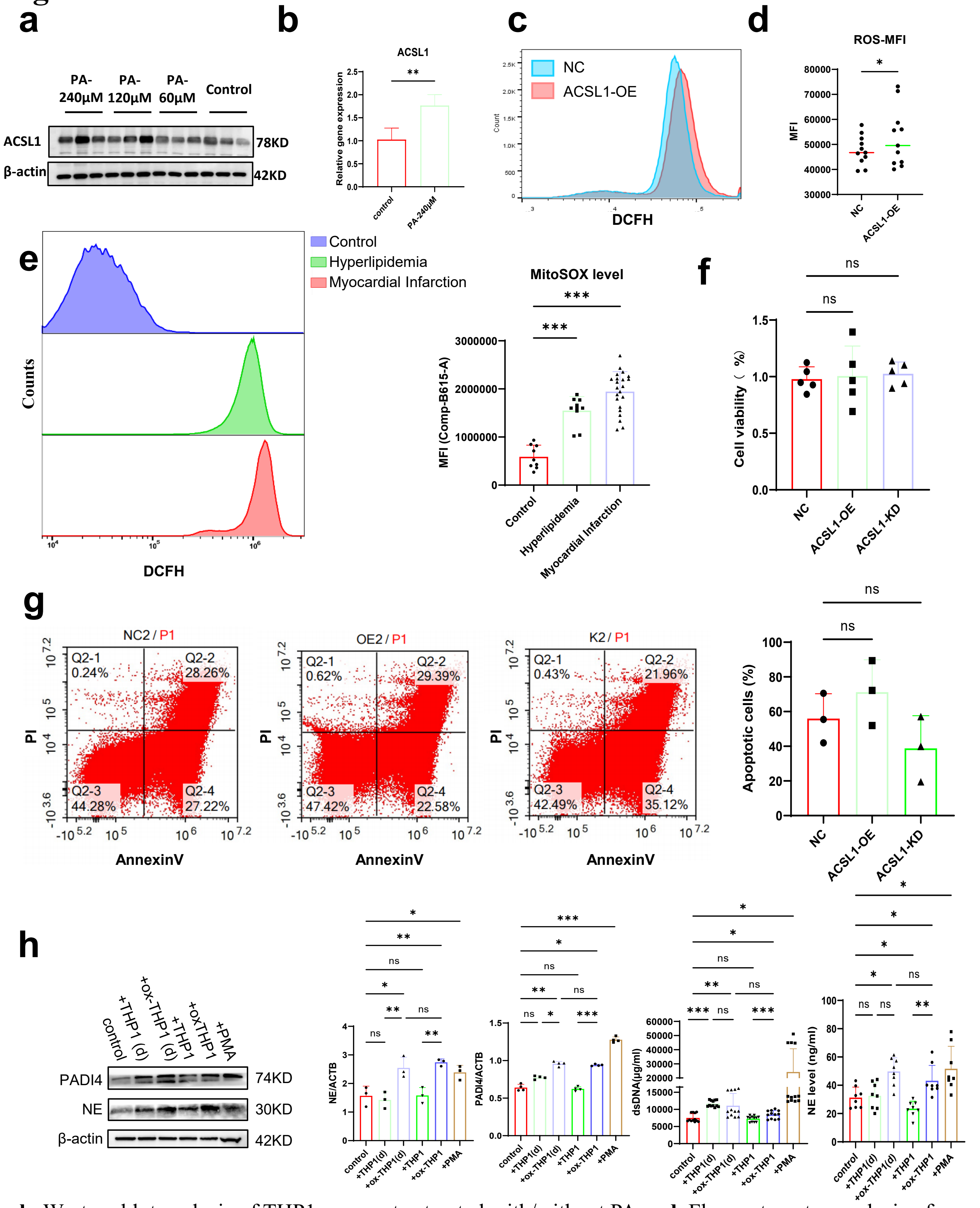

a-b. Western blot analysis of THP1 monocytes treated with/without PA. **c-d.** Flow cytometry analysis of reactive oxygen species (ROS) generation in ACSL1 overexpressed THP1 monocytes. **e.** Flow cytometry analysis of reactive oxygen species (ROS) generation of neutrophils by DCFH and MytoSox. **f-g.** Neutrophils were pre-treated with CM derived from ACSL1-overexpressed-THP1 monocytes or control THP1 monocytes (3 h). Cell viability and apoptosis rate of neutrophils treated with CM from ACSL1-overexpressed. **h.** The release of dsDNA and elastase level from neutrophils treated with CM from or cocultured with THP1 monocytes, oxLDL-pretreated monocytes. The data showed as means ± S.D; one-way ANOVA. *P < 0.05, **P < 0.01, ***P < 0.001, ****P < 0.0001, NS: no significance.

Figure. S6

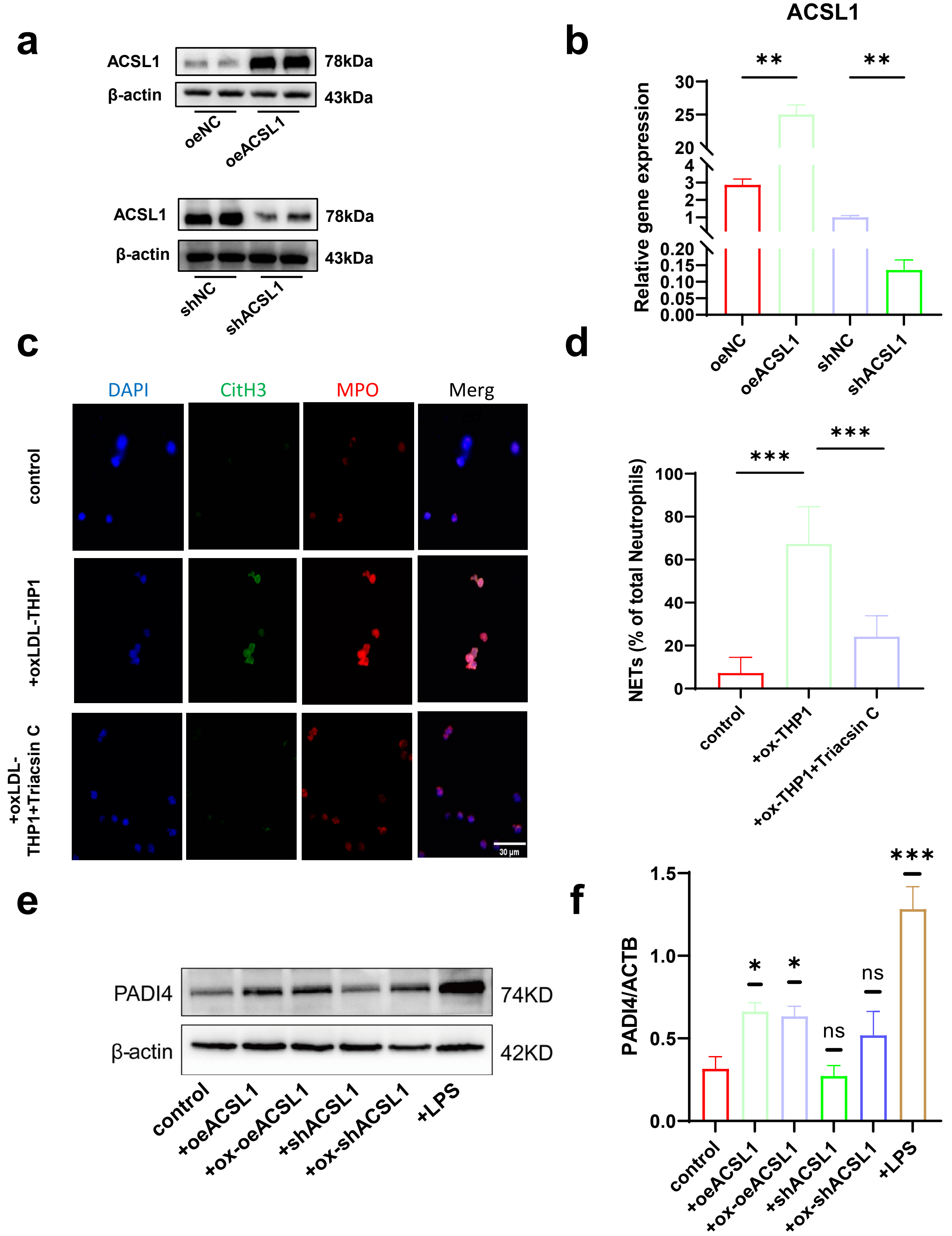

a. Western blot analysis of ASCL1 stable overexpressed (oeACSL1) and knockdown (shACSL1) THP1 cell line. **b.** RT-PCR results of the expression levels of ACSL1 cell lines. **c.** Representative images of immunofluorescence staining showing CitH3 (green) and MPO (red), with DAPI (blue), in peripheral neutrophil cells. Cells were either pre-treated with Triacsin C for 30 minutes or co-cultured with THP1 monocytes pre-treated with or without 100 μg/ml oxLDL. **d.** Quantification of the proportion of NETosis in all neutrophils. (d, n=10 in each group; f, control, n=12; +ox-THP1, n=17; +ox-THP1+Triacsin C, n=11; mean±S.D;). **e-f.** Knocking down ACSL1 in co-culture with THP1 cells can mitigate the activating effect of oxLDL on neutrophils. *P < 0.05, **P < 0.01, ***P < 0.001, ****P < 0.0001, NS: no significance by one-way ANOVA with Tukey post hoc test.

Figure. S7

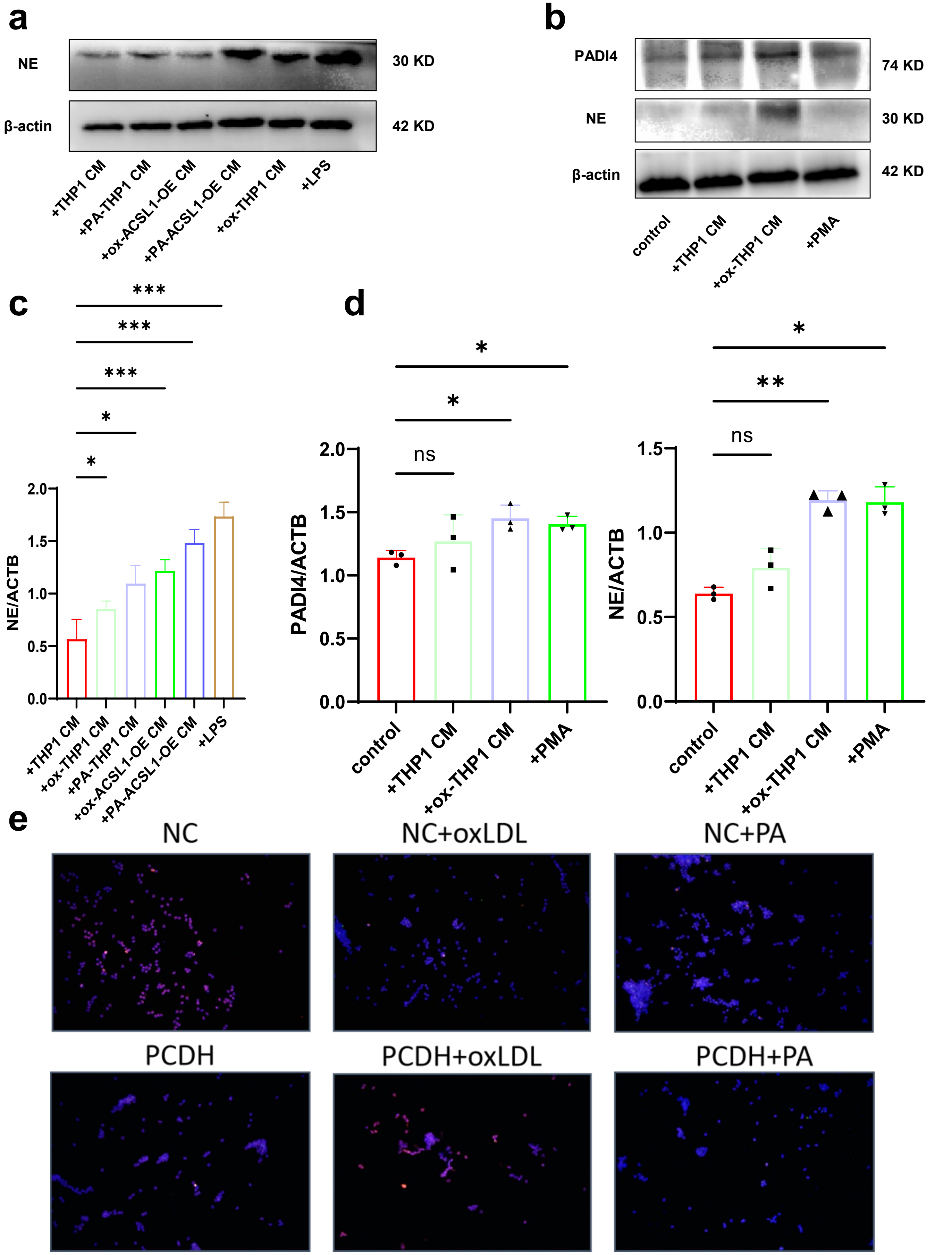

a-d. Western blot analysis of neutrophils treated with CM from PA/oxLDL pre-treated THP1 monocytes and ACSL1-overexpressed THP1 monocytes yielded results similar to those obtained from their coculture. **e.** Immunofluorescence images demonstrating the increase in neutrophil extracellular traps (NETs) following co-culture with oeACSL1 THP1 cells or stimulation with PA/oxLDL. (n=3 each group; The data showed as means ± S.D; one-way ANOVA). $*P < 0.05$, $**P < 0.01$, $***P < 0.001$, $****P < 0.0001$, NS: no significance.

Figure. S8

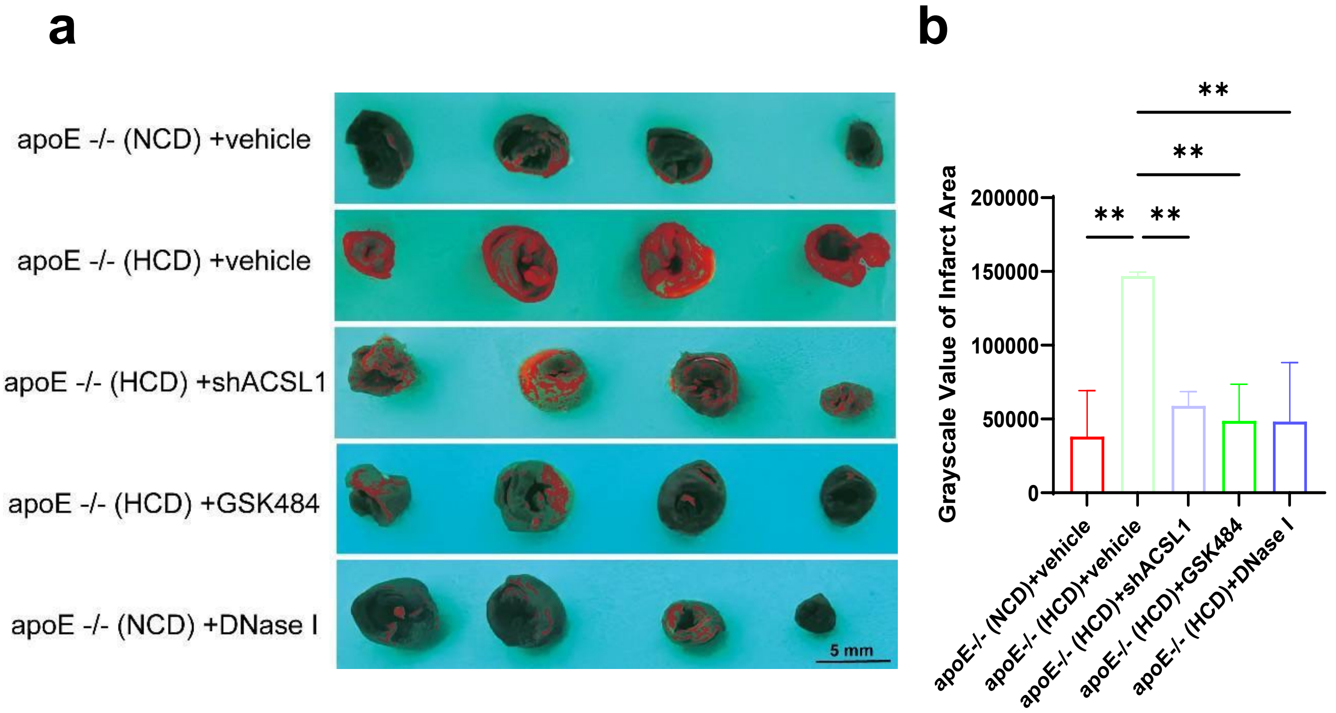

Representative 2, 3, 5-triphenyl-tetrazolium chloride stained serial heart sections from one mouse of each group after 60 minutes of ischemia and 23 hours of reperfusion. **a.** Panels show representative TTC-stained images where the red areas indicate infarct regions in the apoE$^{-/-}$ (NCD)+vehicle group, apoE$^{-/-}$ (HCD)+vehicle group, apoE$^{-/-}$ (HCD)+shACSL1 group, apoE$^{-/-}$ (HCD)+DNase I group, and apoE$^{-/-}$ (HCD)+GSK484 group. **b.** panels show quantification of grayscale value of infarct area in apoE$^{-/-}$ (NCD)+vehicle group, apoE$^{-/-}$ (HCD)+vehicle group, apoE$^{-/-}$(HCD)+shACSL1, apoE$^{-/-}$(HCD)+GSK484, apoE$^{-/-(}$HCD)+DNase I. (n=5 each group; The data showed as means ± S.D; one-way ANOVA). *P < 0.05, **P < 0.01, ***P < 0.001, ****P < 0.0001, NS: no significance.

Figure. S9

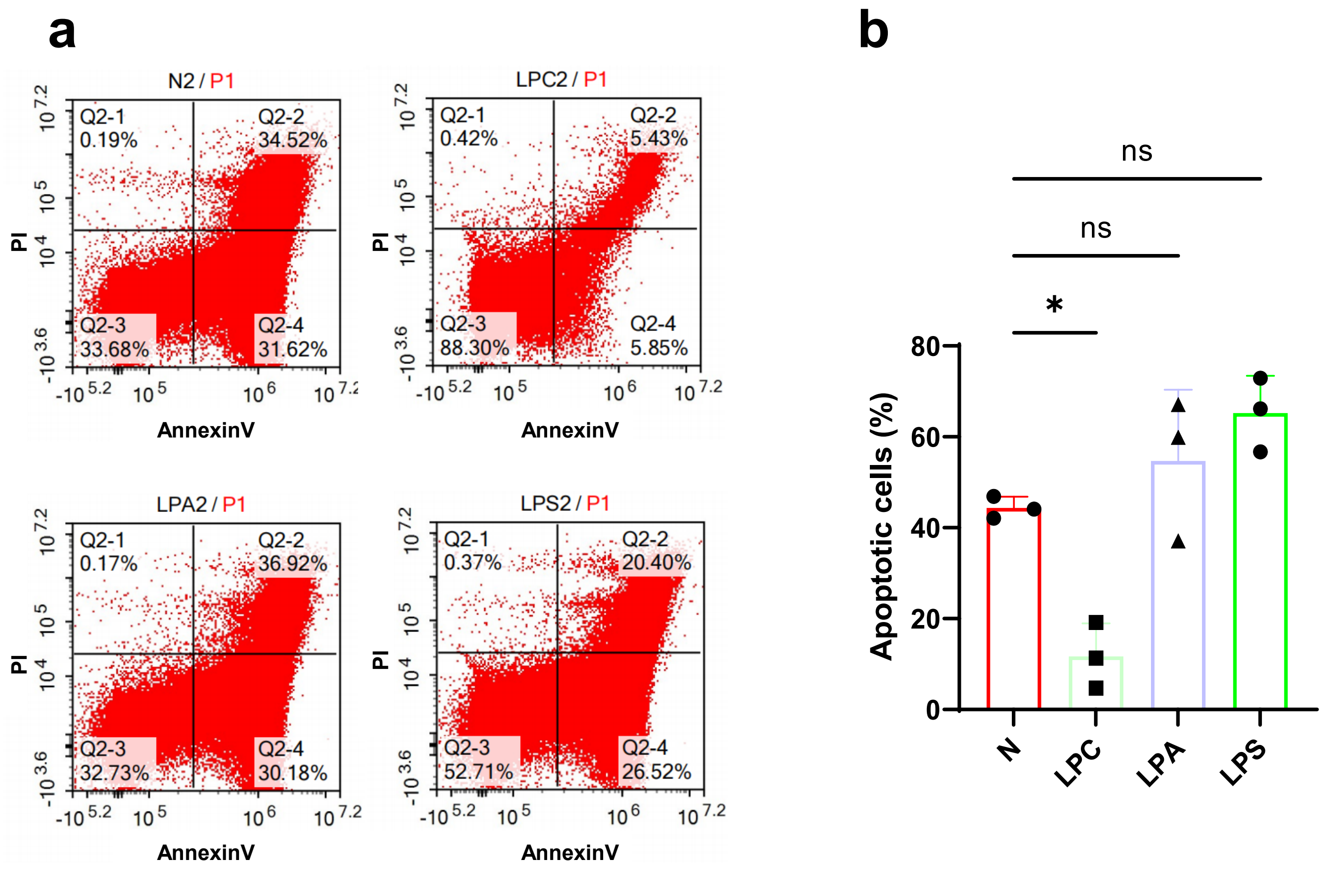

a-b. Flow cytometry Annexin V/PI staining for measuring apoptosis after 3h exposure of neutrophils to the 80 μM LPA/LPC. (n=3 each group; The data showed as means ± S.D; one-way ANOVA). *P < 0.05, NS: no significance.

Figure. S10

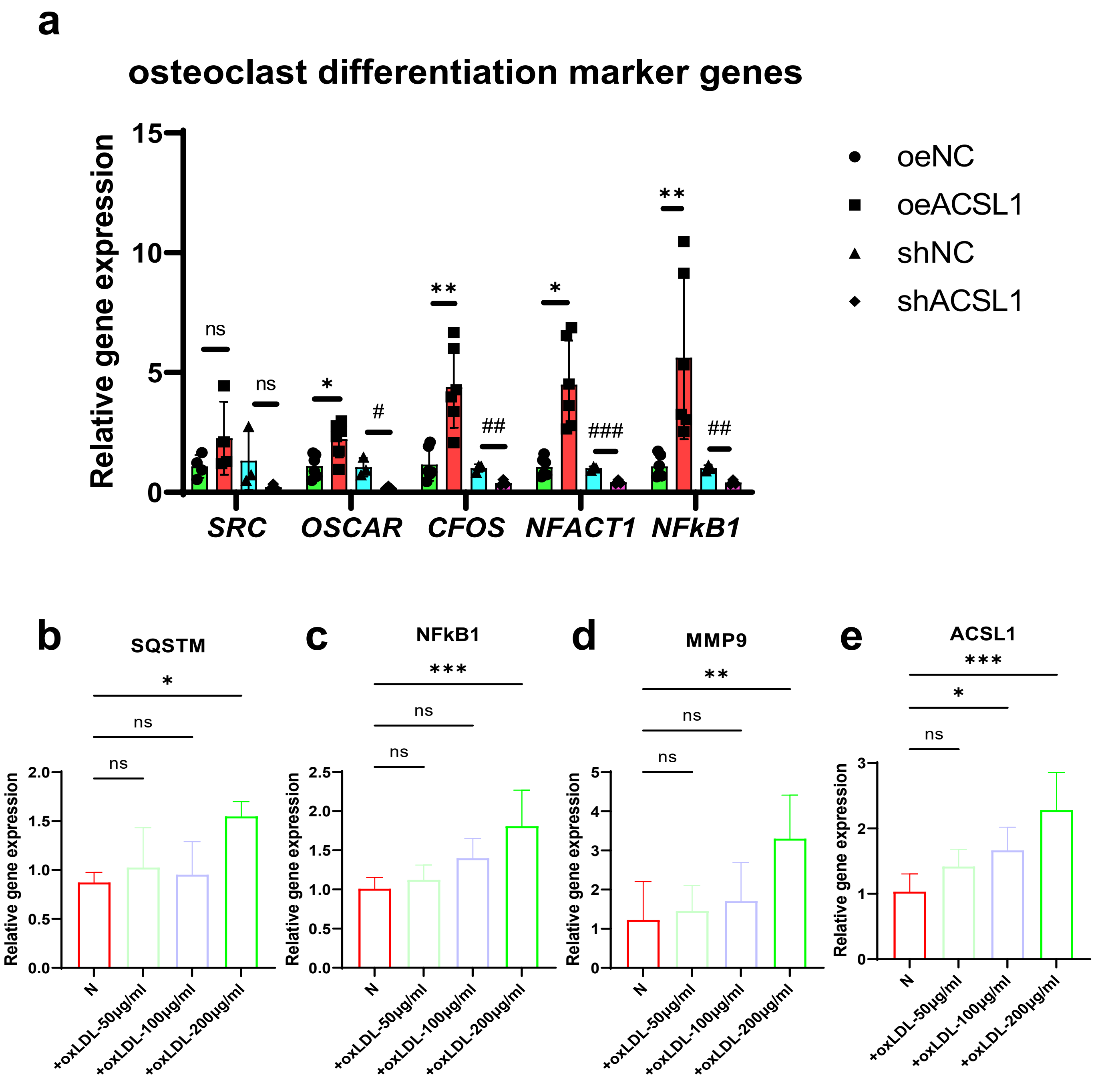

a. Relative fold change of cytokines associated with osteoclast-like cell differentiation measured in ACSL1-overexpressing THP1 monocytes. n=5; *oeNC vs. oeACSL1; #shNC vs. shACSL1. **b-e.** The relative fold change in osteoclast differentiation-related cytokines was measured in oeACSL1 THP1 monocytes. This fold change was first normalized against β-actin and then expressed as a change relative to the expression levels in control THP1 monocytes. n=3; The data showed as means ± S.D; one-way ANOVA. *P < 0.05, **P < 0.01, ***P < 0.001, ****P < 0.0001, NS: no significance.

Figure. S11

Figure 3A

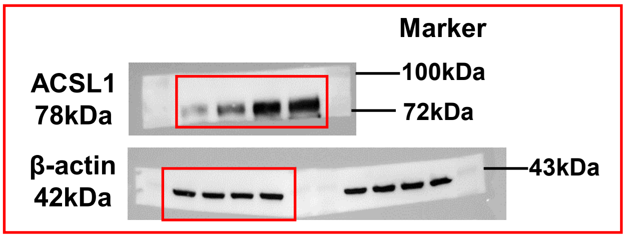

Figure 3C

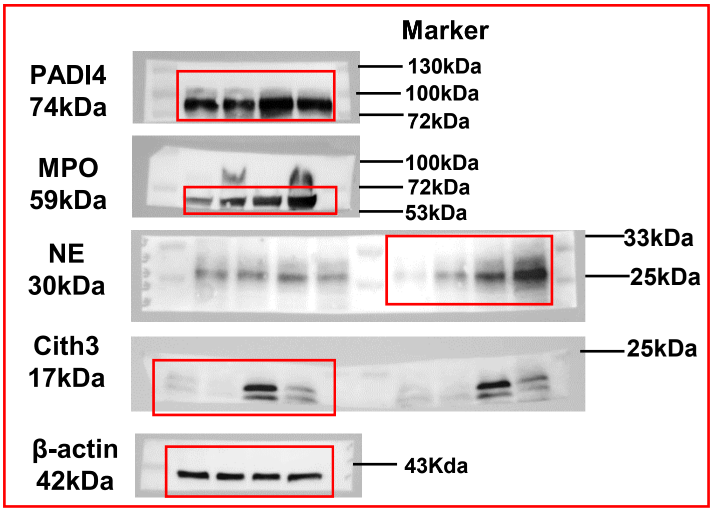

Fig 3E

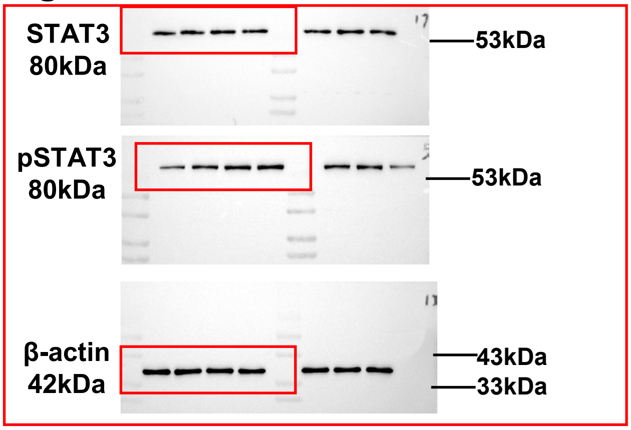

Fig 6D

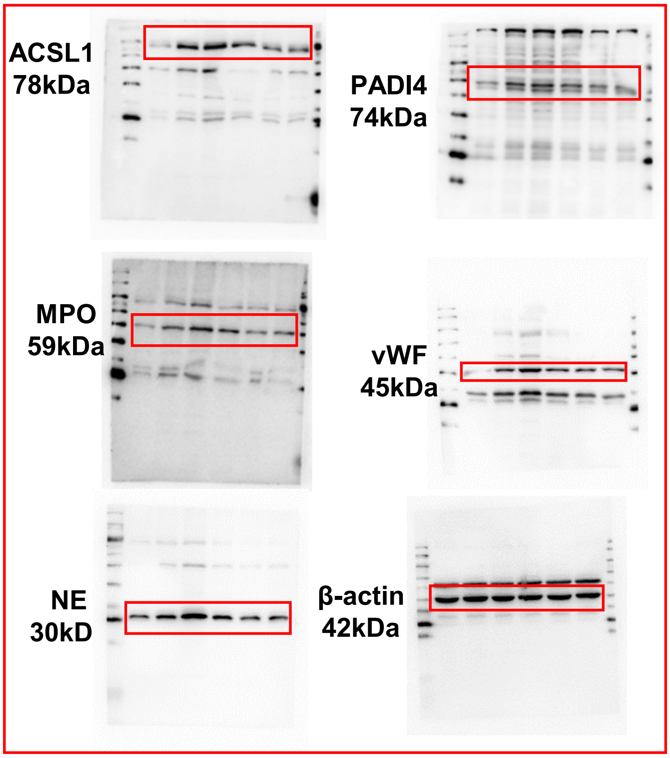

Fig 5C

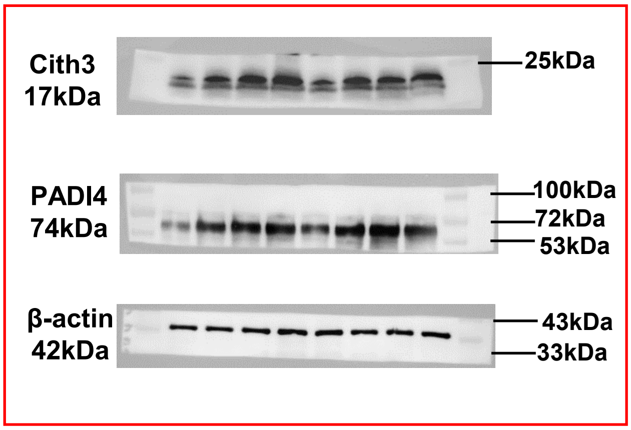

All uncropped images of the western blots in the figures.

Table S1. General Characteristics Data of clinical samples

	Healthy Volunteers N = 77	Hyperlipidemia patients N = 75	AMI patients N = 69
Females/males	41/36	39/36	42/27
Ages, years (range)	67.6±14.0 (42.0-86.0)	62.8±11.0 (44.0-82.0)	75.8±9.1 (58.0-88.0)
Hypertention	30(39.0%)	43(57.3%)	47(68.1%)
Diabetes	2(2.5%)	7(9.3%)	9(13.0%)
Anticoagulants	_	_	20(29.0%)
Ca Channel blockers	_	_	14(20.3%)
Statins	_	_	18(26.1%)
Diuretics	_	_	5(7.2%)
Beta blockers	_	_	14(20.3%)
Angiotensin receptor antagonists	_	_	12(17.4%)
ACE inhibitors	_	_	15(21.7%)

Table S2. Main antibodies and reagents utilized in this study

Antibodies/Reagents	Source	Company	Catalog numbers
Anti-Histone H3	Rabbit	Abcam	ab281584
Anti-Myeloperoxidase	Rat	Abcam	ab300650
Anti-Myeloperoxidase	Rabbit	Abcam	ab208670
Anti-ACSL1	Rabbit	ABclonal	A16253
Anti-Neutrophil Elastase	Rabbit	Affinity	AF0010
Anti-PADI4	Rabbit	Abcam	ab208670
Anti-vWF	Rabbit	Cell signaling technology	65707
STAT3	Rabbit	Cell signaling technology	12640S
p-STAT3	Rabbit	Cell signaling technology	9145S
Ly-6G Rat mAb (FITC)	Rat	Cell signaling technology	68590S
Anti-CD41	Rabbit	ABclonal	A11490
FITC-MPO antibody		Bioss	bsm-30232M
PE/Cy7 Ly-6G antibody		BioLegend	127617
PerCP-CD16 antibody		BioLegend	101230
APC anti-mouse CD45		BioLegend	103112
APC/Cy7 human CD45		BioLegend	304014
BV421 rabbit lgG		BioLegend	406410
Rat Mouse Ly-6B.2 Alloantigen	Rat	BIO RAD	MCA771GA
β-Actin Mouse mAb	Mouse	ABclonal	AC043
Anti-Rabbit IgG H&L (HRP)	Goat	Abcam	ab6721
Goat Anti-Rat IgG H&L (Alexa Fluor® 488)	Goat	Abcam	ab150157
HA130	_	Beyotime	SD7225
Oleoyl-ʟ-α-lysophosphatidic acid sodium salt	_	Sigma	L7260
1-Palmitoyl-sn-glycero-3-phosphocholine	_	Sigma	L5254
DNase I	_	Roche	11284932001
18:1 Lyso PE	_	Sigma	846725P
GSK484	_	MedChemExpress(MCE)	HY-100514
oxLDL	_	Yiyuan Biotechnology Co., Ltd	YB-002
Palmitic acid	_	Sigma	P0500
Triacsin C	_	Cayman	10007448
AM966	_	MedChemExpress(MCE)	HY-15277
Lipopolysaccharides	_	Sigma	L5293
Picogreen dsDNA Quantitation Reagent		yeasen	12641ES02
Calf Thymus DNA Solution		yeasen	60613ES70
SYTOX™ Deep Red		ThermoFisher	S11380

Table S3. Primer sequence for RT-PCR

Gene	Species	Forward (5'-→-3')	Reverse (5'-→-3')
ACTB	human	GCCGACAGGATGCAGAAGGAGATCA	AAGCATTTGCGGTGGACGATGGA
ACSL1	human	ATCAGGCTGCTCATGGATGACC	AGTCCAAGAGCCATCGCTTCAG
MMP9	human	GCCACTACTGTGCCTTTGAGTC	CCCTCAGAGAATCGCCAGTACT
NFKB	human	GCAGCACTACTTCTTGACCACC	TCTGCTCCTGAGCATTGACGTC
OSCAR	human	GCAGCGAGGTGCTGGTCATCA	ACTGCGCCAGTCAAAAGTGACC
CFOS	human	GGGGCAAGGTGGAACAGTTAT	CCGCTTGGAGTGTATCAGTCA
PLA1A	human	GTGTGGAAGAGCGCTTGGAT	GGCCTCCGTTGACGAAGTAG
PLA2G4A	human	GTGATGTGCCTGTGGTAGCC	CCAGCAACGTAGGTAGCACAAT
ENPP2	human	ACGTGGAAGGCAGTTCCATT	GGCAGGATGAAGGAGGACAC
SQSTM1	human	TGTGTAGCGTCTGCGAGGGAAA	AGTGTCCGTGTTTCACCTTCCG
Nfatc1	human	CACCAAAGTCCTGGAGATCCCA	TTCTTCCTCCCGATGTCCGTCT
SREBP1	human	CGGAACCATCTTGGCAACAGT	CGCTTCTCAATGGCGTTGT
PTPMT1	human	ATCGCCAAGATCCGGTCATAC	CAGTAATCTGCTTGTGGAACTCT
GPD1	human	GCCATCTGAAGGCAAACGC	GCCAATGGTTGTCTCACAGAAC
LPIN1	human	CCAGTGTAGTCCAGACAGCAAACAA	TAGACGCCGTCAGCACCAAG
LPIN2	human	GGACCCACCAGGGTATAGCAAAG	TGTCATTGACCCAGTGCAGGTAG
AGPAT2	human	CCGTGGTGTACTCTTCCTTCTCCT	CAGCACCTGCACTGTGACTGTT
AGPAT4	human	CGAACGCTTTGGGCTGTTAG	TGAAGTACCACATCCAGCCGATA
GPAT1	human	GTTCTGCCCAGCAGCAATCA	TTTAGCAGCACCCACCCAGTC
GPAT4	human	TGTGGTCGTCAGGCTTTGG	GGTCCGTTATGCTTCTGTGGA
SCD1	human	AAACCTGGCTTGCTGATG	GGGGGCTAATGTTCTTGTCA
PPARG	human	GCTCCGTGGATCTCTCCGTA	TCTGCAACCACTGGATCTGTTC
FADS3	human	AGAACCCAGCCAGGATGGAC	CAGGATGTGGCCCAGTAGGAA
CDS2	human	TTCGCAAGTGGATTCAAACGAG	CATTGACAAAGGTGGCCATCAG
CRLS1	human	AGAGATGTAATGTTGATTGCTGCTG	TGCCACCAAGATTAACTGGACTG
ACC	human	ATGTCTGGCTTGCACCTAGTA	CCCCAAAGCGAGTAACAAATTCT
FAS	human	AAGGACCTGTCTAGGTTTGATGC	TGGCTTCATAGGTGACTTCCA
SRC	human	CTGCTTTGGCGAGGTGTGGATG	CCACAGCATACAACTGCACCAG
CTSK	human	ACTCAAAGTACCCCTGTCTCAT	CCACAGAGCTAAAAGCCCAAC
